# Endoplasmic reticulum-resident protein DNAJC10 inhibits glioblastoma metastasis by suppressing XBP-1s-driven *EGFR* transcription

**DOI:** 10.1186/s43556-025-00308-0

**Published:** 2025-10-24

**Authors:** Erdi Zhao, Yue Yu, Yingli Gao, Teng Li, Shiyu Hao, Meiyang Chen, Ming Xu, Sinkemani Arjun, Chunyan Yang, Yancun Yin, Minjing Li

**Affiliations:** 1 Laboratory of Experimental Hematology, School of Basic Medical Sciences, Binzhou Medical University, Yantai, 264003 China; 2 The Second School of Clinical Medicine, Binzhou Medical University, Yantai, 264003 China; 3 Institute of Stomatology, Binzhou Medical University, Yantai, 264003 China; 4 The affiliated Yantai Stomatological Hospital, Binzhou Medical University, Yantai, 264003 China; 5 School of Traditional Chinese Medicine, Binzhou Medical University, Yantai, 264003 China

**Keywords:** Glioblastoma, Metastasis, EGFR, Tumor suppressor, Unfolded protein response (UPR)

## Abstract

Glioblastoma (GBM) is characterized by the highly infiltrative growth of cancer cells into the surrounding brain parenchyma. DnaJ Heat Shock Protein Family (Hsp40) Member C10 (DNAJC10, also known as ERDJ5 and PDIA19), involved in endoplasmic reticulum-associated degradation (ERAD), has been identified as a tumor suppressor in several cancers. However, its precise role and underlying mechanism in GBM remain unclear. We found that DNAJC10 expression is downregulated in GBM patients and correlated with poor survival outcomes. Overexpression of *DNAJC10* reduced GBM cell migration and invasion in vitro, while its knockdown promotes these processes. Moreover, *DNAJC10* overexpression inhibits infiltrative growth of GBM cells, suppresses tumor propagation and prolongs survival in xenografted mice. Mechanistically, DNAJC10 regulates multiple molecules and pathways involved in cell motility, including the epidermal growth factor receptor (EGFR) pathway. Importantly, DNAJC10 overexpression decreases *EGFR* transcription by inhibiting spliced X-box binding protein 1 (XBP-1s). DNAJC10 regulates XBP-1s splicing through the inositol-requiring enzyme 1α (IRE1α) branch of the unfolded protein response (UPR). XBP-1s binds the EGFR promoter and enhances recruitment of SET7/9 methyltransferase, H3K4me3, and H3K4me1. Pharmacological inhibition of histone methylation attenuates XBP-1s-induced *EGFR* transcription, indicating XBP-1s promotes *EGFR* expression via recruiting SET7/9 for H3K4 methylation. XBP-1s overexpression reverses DNAJC10-mediated *EGFR* downregulation. Collectively, DNAJC10 suppresses *EGFR* transcription by inhibiting the UPR IRE1α-XBP-1s axis, reducing SET7/9 recruitment and H3K4 methylation at the *EGFR* promoter. Targeting DNAJC10 or XBP-1s could be a potential approach for inhibiting GBM infiltration and may represent a novel avenue for GBM treatment.

**Supplementary Information:**

The online version contains supplementary material available at 10.1186/s43556-025-00308-0.

## Introduction

Glioblastoma (GBM) is the most aggressive form of primary malignant tumor in the central nervous system, with a median survival of only 14–16 months and an overall 5-year survival rate at about 5% [1, 2]. Despite advances in surgery, chemotherapy, and radiotherapy, its prominent clinicopathological feature—diffuse infiltration of cancer cells into the surrounding brain parenchyma—makes complete surgical resection nearly impossible without severe neurological damage [3]. This infiltration is a major barrier to curative therapies, driving tumor recurrence and poor prognosis [2]. Therefore, identifying molecular targets regulating GBM invasion/migration is critical for developing effective therapies, but the underlying mechanisms remain poorly understood [4].

Accumulating evidence links the unfolded protein response (UPR) to cancer cell invasion and migration [5]. UPR sensors activating transcription factor 6 (ATF6), protein kinase R-like endoplasmic reticulum kinase (PERK) and inositol-requiring enzyme 1α (IRE1α) and their down-stream pathways regulate processes involved in cell invasion or migration such as extracellular matrix (ECM) remodeling [6], cytoskeleton reorganization [7], epithelial-to-mesenchymal transition (EMT) [8, 9]. Notably, heat shock protein family members (e.g., DNAJA4 [10], HSPB7 [11]) and endoplasmic reticulum (ER) stress-related proteins [12, 13], modulate migration and invasion in various cancers [12–15], highlighting their potential role in GBM motility.

DNAJC10 is an endoplasmic reticulum (ER)-resident protein with one J domain and six thioredoxin domains. It participates in endoplasmic reticulum associated degradation (ERAD) by recognizing misfolded proteins, reducing incorrect disulfide bonds, and cooperating with GRP78 and EDEM1 to maintain ER homeostasis [14–16]. Given its role in regulating protein quality control, DNAJC10 balances cell survival and apoptosis, influencing cancer progression [17, 18]. It acts as a tumor suppressor in prostate [19], neuroblastoma [20], and colon cancer [21]. Downregulation of *DNAJC10* mRNA is associated with poor survival in breast cancer [22]. However, its role in GBM, particularly in regulating invasion and migration, remains unreported [23].

Epidermal growth factor receptor (EGFR), overexpressed in 40%−50% of GBM cases[24], is considered to be a key gene for GBM pathogenesis. Expression of *EGFR* is regulated by a 5’-regulatory sequence, which contains a GC rich promoter without TATA or CAAT boxes [25]. Therefore, basal transcription of *EGFR* gene is regulated by the transcription factor SP1 and starts at multiple initiation sites within the promoter region [25]. However, the transcriptional regulation of *EGFR* gene in cancer cells remains largely unknown.

In this study, we aim to elucidate the role and molecular mechanism of DNAJC10 in GBM invasion and metastasis. We found that DNAJC10 appears to act as a novel tumor suppressor gene in GBM. Overexpression of *DNAJC10* inhibits motility, and tumorigenicity of GBM. Moreover, XBP-1s, a downstream target of DNAJC10, may regulate transcription of *EGFR*, suggesting that DNAJC10/XBP-1s/EGFR axis could be a significant target aimed at destructing migration or metastasis of GBM cells.

## Results

### Low expression of DNAJC10 is associated with the poor prognosis of GBM patients

**Fig. 1:**
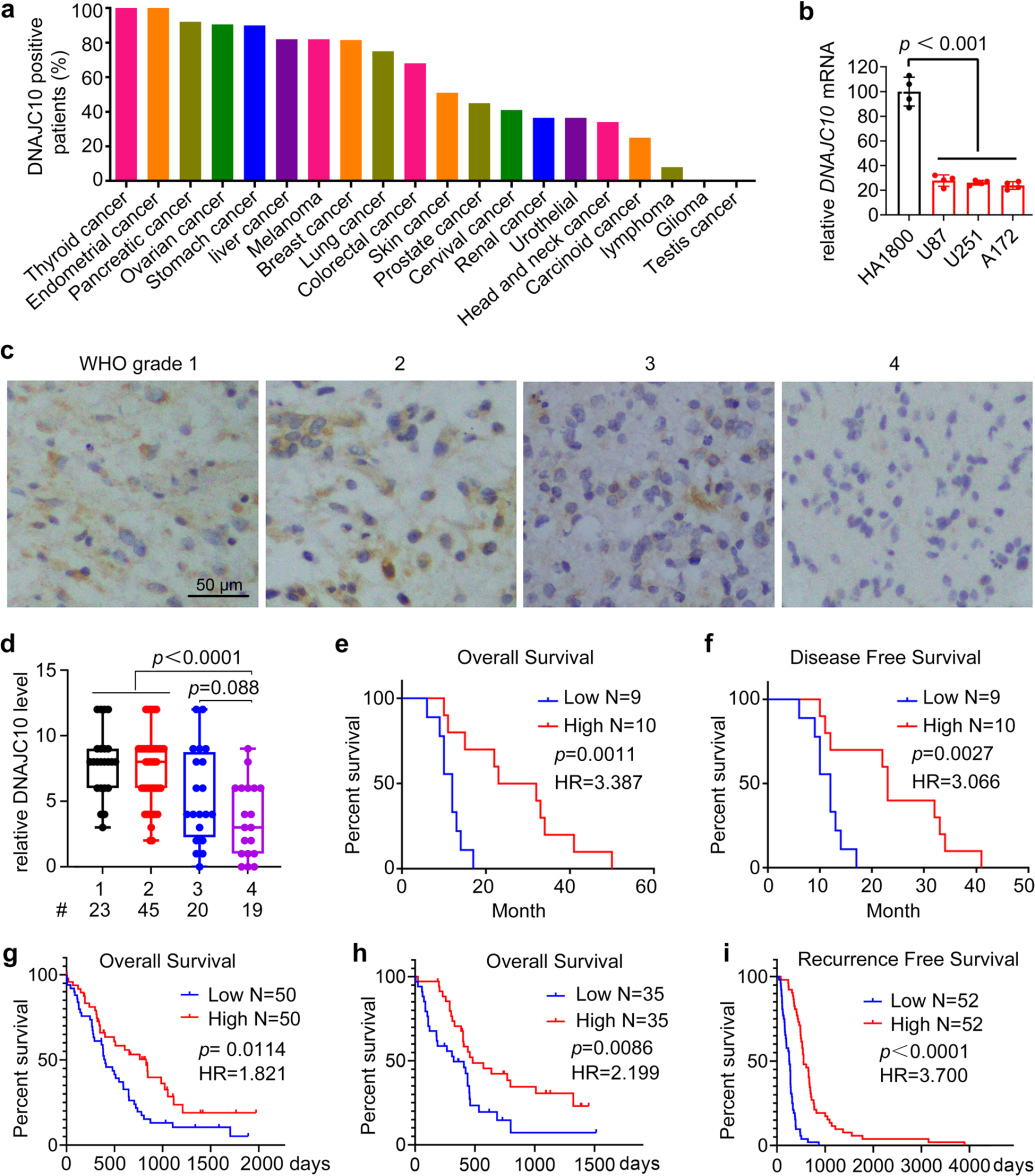
The expression of DNAJC10 positively correlates with survival of GBM patients. **a** The positive expression rate of DNAJC10 in different cancers was analyzed by IHC. Weak staining or not detected samples are defined as negative. Data from the Human Protein Atlas: HPA031111. **b** RT-qPCR analysis of transcription of *DNAJC10* in GBM cell lines and normal human glial cell line HA1800. (GAPDH used as the internal reference gene). **c**,** d** IHC analysis of expression of DNAJC10 in glioma patients with different WHO grades (GBM microarray: #HBraG177Su01; #, patient sample size). Values represent the median score of three raters. Fleiss' *κ* = 0.799 (95% CI: 0.75–0.85). **e-i** Kaplan-Meier survival curves of patients with low or high DNAJC10 expression. Patients were separated into two groups equally based on the median DNAJC10 expression.g-i, data from CPTAC-GBM database. log-rank (Mantel-Cox) test

To determine DNAJC10’s role in glioma, we first assessed its expression in clinical specimens using the Human Protein Atlas (HPA). DNAJC10 was highly expressed in most cancers but largely undetected in gliomas (Fig. 1a). Consistently, RT-qPCR confirmed significantly lower *DNAJC10* mRNA levels in GBM cell lines than in the normal human glial cell line HA1800 (Fig. 1b). Immunohistochemistry (IHC) of 107 clinical brain specimens revealed DNAJC10 was largely undetected or reduced in GBM (WHO grade 4) samples, but prevalent in low-grade gliomas (Fig. 1c,d and Table S2). These observations suggest DNAJC10 downregulation in GBM. We further assessed DNAJC10’s correlation with clinical outcomes. Kaplan–Meier analysis in 19 GBM patients linked low DNAJC10 protein expression to worse overall survival (OS) and disease-free survival (DFS) (Fig. 1e,f). To address small sample size limitations, we expanded via the #HBraG177Su01 microarray dataset (including low-grade gliomas), which showed lower DNAJC10 correlated with poorer survival across all glioma grades (Fig. S1a). Additionally, Clinical Proteomic Tumor Analysis Consortium (CPTAC) proteogenomic GBM dataset confirmed higher DNAJC10 protein levels correlated with longer OS and recurrence-free survival (RFS) (Fig. 1g-i). The Cancer Genome Atlas Program (TCGA) GBM datasets further showed decreased *DNAJC10* mRNA linked to shorter OS and DFS (Fig. S1b,c). Taken together, these observations confirm low DNAJC10 expression is closely associated with poor prognosis of GBM patients.

### DNAJC10 inhibits migration and invasion of GBM cell

**Fig. 2:**
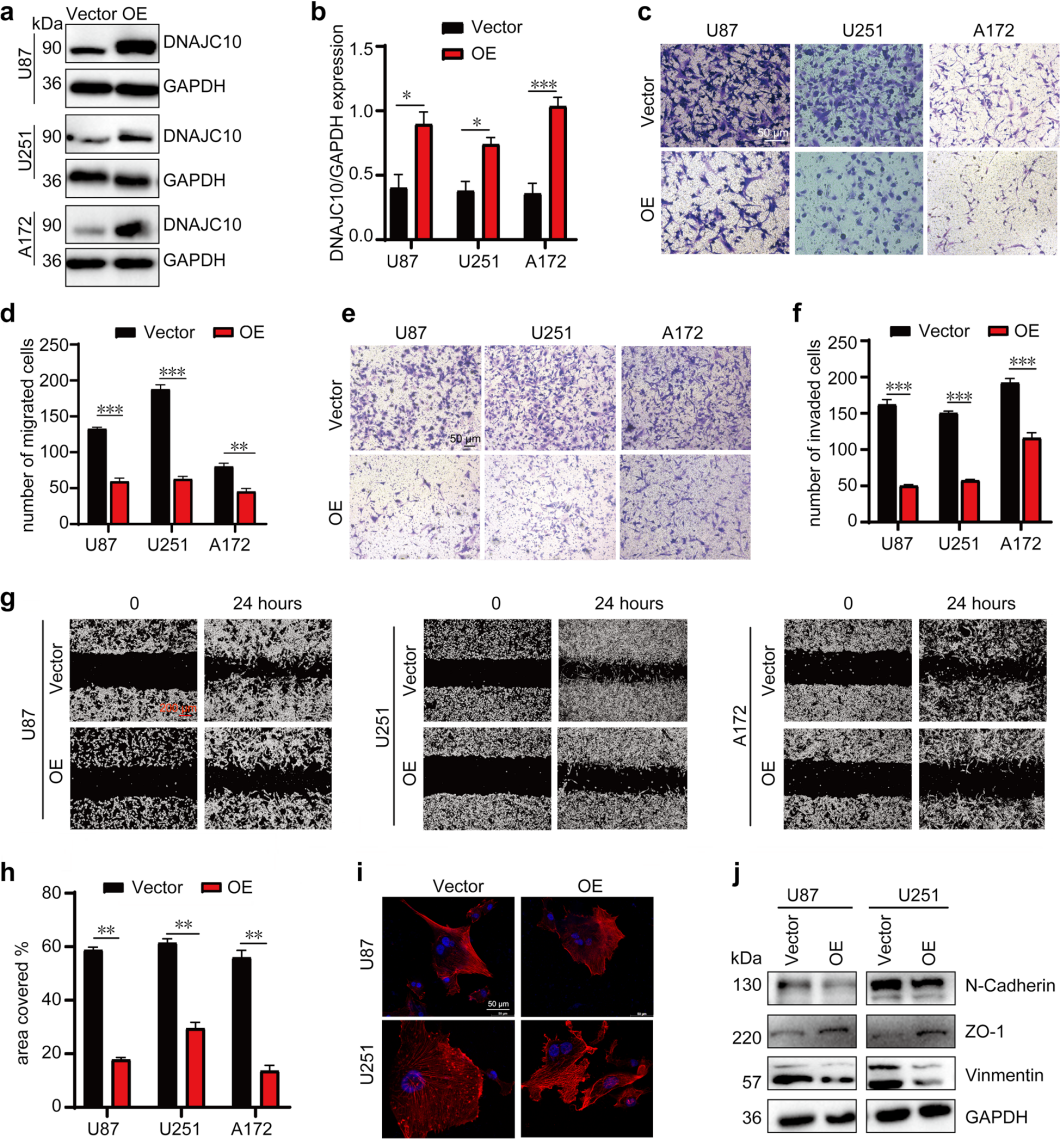
Overexpression of DNAJC10 inhibits migration and invasion of GBM cells. **a **Western blot analysis expression of DNAJC10 in the indicated cell lines infected with Vector or *DNAJC10*-OE lentivirus. **b** Statistical analysis of panel A (*N* = 3). **c-f** Trans-well assays were performed to compare migration (chamber without matrix glue) and invasion (chamber coated with matrix glue) ability in the Vector or *DNAJC10*-OE lentivirus infected cells. Representative images of cell migration (c) and cell invasion (d). The number of migrated cells (e) and invaded cells (f) per field (*N* = 4). **g, h** Scratch wound assay was performed to evaluate Cell migration ability. g, Representative images are shown; h, covered wound area was analyzed in panel g (*N* = 4). **i** the cytoskeleton was visualized by Rhodamine-labeled phalloidin immunofluorescence assay. **j** Western blot analysis the expression of the EMT related proteins in Vector or *DNAJC10*-OE cells. (All the data are from 3 independent experiments. Multiple t-test *, *p* < 0.01; **, *p* < 0.001; ***, *p* < 0.0001.)

Invasion and migration drive poor prognosis and treatment resistance in GBM. To determine DNAJC10’s role in these processes, we overexpressed it in three GBM cell lines with relatively low DNAJC10. Western blot confirmed stable DNAJC10 overexpression in U87, U251 and A172 cells (Fig. 2a,b). Next, trans-well assays assessed DNAJC10’s effects on GBM cell migration and invasion. *DNAJC10* overexpression significantly decreased the number of migrating and invading cells compared to the Vector control group (Fig. 2c-f). Additionally, wound healing assays also revealed reduced cell migration across various GBM cell lines upon *DNAJC10* overexpression (Fig. 2g,h). DNAJC10 overexpression did not affect GBM cell proliferation (Fig. S2), confirming that observed migration phenotypes in wound healing and trans-well assays were due to altered motility, not proliferation.

To confirm effect specificity, loss-of-function experiments using distinct shRNA constructs targeting DNAJC10 were performed. DNAJC10 knockdown consistently enhanced cell migration and invasion in a manner reciprocal to overexpression phenotypes (Fig. S3). This bidirectional regulation (suppression via overexpression and promotion via knockdown) confirms DNAJC10 inhibits GBM cell migration and invasion. To assess off-target effects on normal cells, we overexpressed *DNAJC10* in the human normal glial cell line HA1800. Notably, no significant differences in cell proliferation, viability, or morphology were observed compared to controls, providing preliminary evidence safety in non-tumorigenic glial cells (Fig. S4). These findings collectively validate DNAJC10 as a specific inhibitor of GBM migration and invasion, with minimal impact on normal cellular functions.

Cytoskeletal remodeling is critical for cell migration and invasion. To examine DNAJC10’s effect on intracellular cytoskeletal, we stained cells using Rhodamine-labeled phalloidin. Immunofluorescence showed significant cytoskeletal disorganization in *DNAJC10*-overexpressing cells compared to control group (Fig. 2i). EMT associated molecules are closely linked to cellular migration, invasion, and cytoskeletal dynamics [26]. Based on immunofluorescence results, we further investigated EMT-related proteins through Western blot. *DNAJC10* overexpression significantly downregulated N-cadherin and Vimentin, and upregulated ZO-1 (Fig. 2j). Together, these results indicate DNAJC10 regulates EMT-related proteins and inhibits GBM cell motility.

### Overexpression of DNAJC10 suppresses GBM invasion and metastasis *in vivo*

**Fig. 3:**
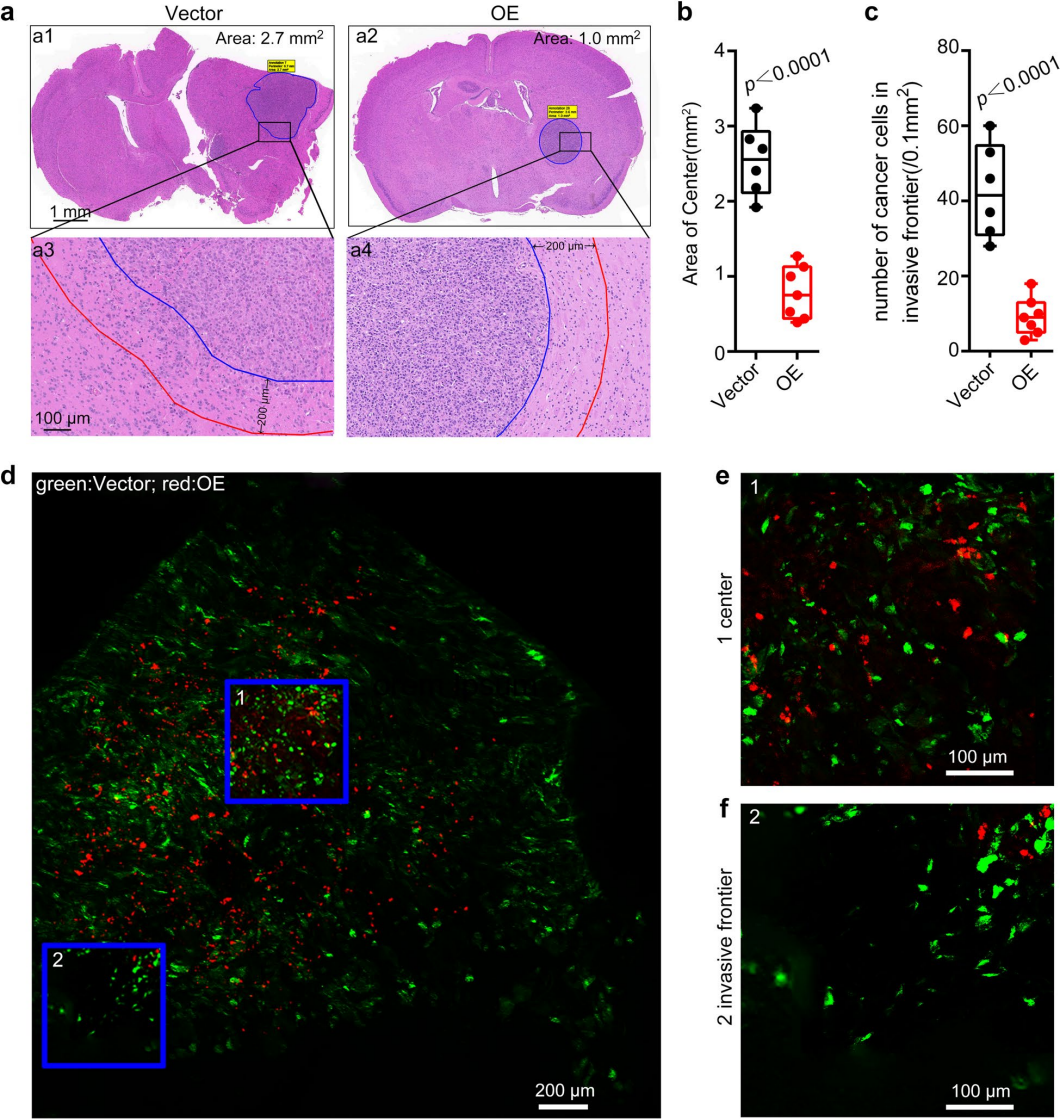
Overexpression of DNAJC10 inhibits GBM cells invasion in vivo. (**a**) H&E staining of brain sections of NSG mouse implanted with U87 cells that infected with Vector or *DNAJC10*-OE lentivirus. (**b**) Quantification of the orthotopic tumor size in panel a1 and a2. The center is the region within the blue line. Student’s t-test, *p* < 0.0001.Vector group *N* = 6; DNAJC10-OE group *N* = 7 (cohen’s d ≥ 1.38, power ≥ 94.29%). (**c**) Quantification of the number of infiltrated cancer cells in the invasive frontier (between the blue and the red line, A3 and A4). Six regions were randomly selected for each mouse to calculate the average value. Student’s t-test, *p* < 0.0001.Vector group *N* = 6; DNAJC10-OE group *N* = 7 (cohen’s d ≥ 1.38, power ≥ 94.29%). (**d**) A mixture of GFP-labeled Vector cells and RFP-labeled DNAJC10-OE cells (Vector:OE = 1:1.5) was injected into the NSG mouse brain. The brain sections of the xenografted mouse was subjected to frozen section and dual-fluorescence at 30 days post-transplantation. Images showed the (**e**) center (**f**) and periphery of the xenografted tumor

To mimic the in vivo tumor microenvironment, an intracranial orthotopic xenograft model was established to assess GBM invasion and metastasis. The hematoxylin & eosin (H&E) staining revealed *DNAJC10*-OE tumors had significantly smaller tumor volumes than the Vector group (Fig. 3a,b). Importantly, *DNAJC10*-OE intracranial tumors exhibited clear, regular round boundaries, whereas Vector tumors had ill-defined boundaries and invaded surrounding brain tissue (Fig. 3a). Further analysis demonstrated *DNAJC10* overexpression significantly reduced cancer cells numbers at the tumour invasive frontier (defined as 200 μm from the tumor edge; Fig. 3c).

Next, we employed an internally controlled dual-fluorescence approach to assess DNAJC10’s impact on GBM invasion and migration in vivo [27]. Dual-fluorescence imaging revealed GFP-labeled Vector cells exhibited wider distribution and greater migratory distances, while RFP-labeled *DNAJC10*-OE cells are tend to localize to the tumor center (Fig. 3d-f). At the tumor invasive frontier, GFP-labeled Vector cells outnumbered RFP-labeled *DNAJC10*-OE cells (Fig. 3f). These results suggeste *DNAJC10* overexpression inhibits GBM invasion and migration in vivo.

### Overexpression of DNAJC10 inhibits GBMprogression and prolongs survival in tumor-bearing mice

**Fig. 4:**
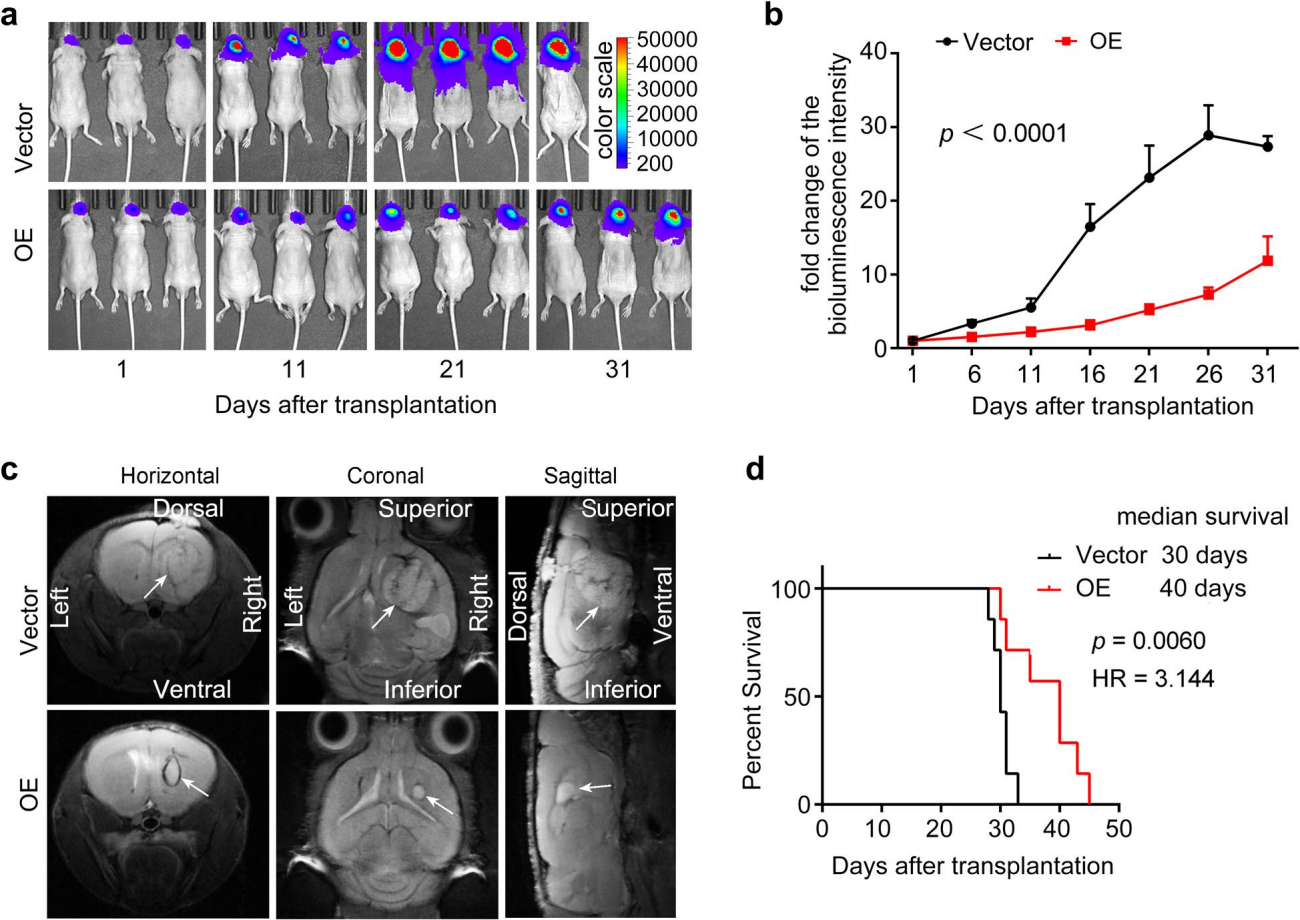
Overexpression of *DNAJC10* inhibits GBM development in vivo. Luciferase-labeled Vector or *DNAJC10*-OE U87 cells were intracranially injected into NSG mice aged 6–8 weeks. The xenografted mice were randomly divided into three groups, which were used for (a-b) bioluminescence imaging, (c) magnetic resonance imaging (MRI), and (d) Kaplan–Meier survival analysis, respectively. (**a**) Representative photon flux images of the xenografted mouse brain. (**b**) Quantification of the luciferase activity with the Living Image software (Vector group *N* = 5; *DNAJC10*-OE group *N* = 5; cohen’s d ≥ 1.38, power ≥ 86.73%). (**c**) Representative MRI images of orthotopic tumor-bearing mice obtained at 30 days after transplantation. (**d**)**,** Overall survival of the xenografted mice was analyzed by Kaplan–Meier and log-rank (Mantel-Cox) test (Vector group *N* = 7; DNAJC10-OE group *N* = 7; cohen’s d ≥ 1.38, power ≥ 98.57%)

To investigate DNAJC10’s effect on GBM development in *vivo*, we established orthotopic xenografts using luciferase-labeled Vector or *DNAJC10*-OE GBM cells. Bioluminescent analysis revealed *DNAJC10* overexpression substantially inhibited GBM growth in the orthotopic model (Fig. 4a,b). The average fluorescence intensity in Vector-cell-injected mice was > fivefold higher than in DNAJC10-OE-cell-injected mice, at 16 days post transplantation (Fig. 4b). Moreover, most Vector-cell-injected mice had marked weight loss by 15 days post transplantation, whereas DNAJC10-OE-cell-injected mice had no obvious weight loss, to some extent indicating *DNAJC10* overexpression inhibits GBM progression in vivo (Fig. S5). Further, magnetic resonance imaging (MRI) demonstrated *DNAJC10*-OE tumors were consistently smaller than Vector tumors. Additionally, *DNAJC10*-OE tumors exhibited well-defined boundaries compared to infiltrative growth of Vector tumors (Fig. 4c). Kaplan–Meier analysis indicated *DNAJC10* overexpression significantly prolonged OS of the xenografted mice. Vector-cell-injected mice survived medium of 30 days, whereas *DNAJC10*-OE-cell-injected mice survived medium of 40 days (Fig. 4d). To address genetic heterogeneity, additional in vivo experiments were conducted using A172 cells harboring the EGFR variant III mutant (EGFRvIII), a common GBM variant linked to therapy resistance. Strikingly, *DNAJC10*-OE A172 xenografts displayed significantly reduced tumor infiltration and prolonged survival (Fig. S6) compared to Vector controls, mirroring results from wild-type EGFR-expressing U87 models. Together, these results demonstrate DNAJC10 overexpression inhibits GBM progression across different EGFR genetic backgrounds, highlighting its robust in vivo tumor-suppressive activity.

### RNA-seq identifies multiple signaling pathways associated with DNAJC10 expression in GBM cells

**Fig. 5:**
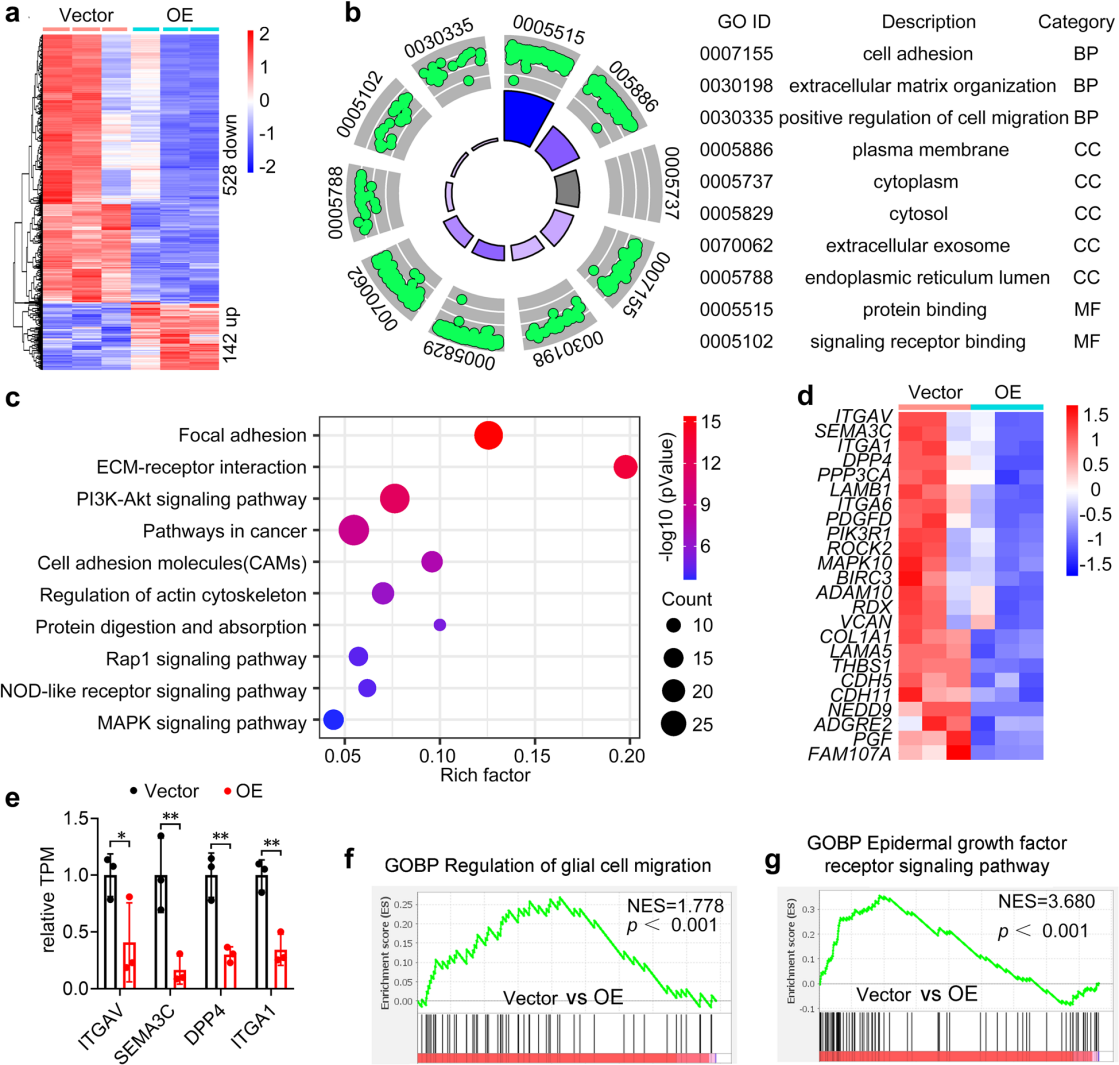
Overexpression of *DNAJC10* regulates multiple signaling pathways in GBM. **a** Heatmap showing the 670 DEGs between Vector and *DNAJC10*-OE cells using RNAseq. (**b**) GO enrichment analyses of the 528 downregulated DEGs. Shown are the most fold enrichment terms in biological process (BP), molecular function (CC), and molecular function (MF). (**c**) The top 10 enriched KEGG pathways of the 670 DEGs in RNAseq. (**d**) Heatmap analysis of the targeted genes in cell adhesion molecules (CAMs) term. The DEGs that positive regulating cell migration and invasion are downregulated in *DNAJC10*-OE group. (**e**) Relative expression TPM values of the indicated CAMs-related genes. Data from RNAseq. Multiple t-test, *, *p* < 0.01; **, *p* < 0.001. (**f-g**) GSEA for regulation of glial cell migration and epidermal growth factor receptor signaling pathway

To identify molecular mechanisms underlying DNAJC10-mediated inhibition of GBM invasion and migration, Vector and *DNAJC10*-OE GBM cells were subjected to RNAseq. 670 differentially expressed genes (DEGs) following *DNAJC10* overexpression were used for further enrichment analysis (Fig. 5a). GO enrichment analysis revealed downregulated DEGs were enriched in multiple functional terms including ER lumen and protein binding (Fig. 5b), aligning with DNAJC10’s known role as a component of ERAD complex[19]. Remarkably, the downregulated DEGs in *DNAJC10*-OE group were also enriched in cell adhesion, ECM organization, and positive regulation of cell migration, suggesting *DNAJC10* overexpression suppresses glioma cell migration (Fig. 5b). Consistently, KEGG analysis revealed DEGs enriched in pathways linked to cell migration and invasion, including ECM-receptor interaction, Cell adhesion molecules (CAMs), and Regulation of actin cytoskeleton (Fig. 5c). Furthermore, Heatmap analysis showed *DNAJC10* overexpression reduced transcription of CAMs related genes such as *ITGAV* [28], *SEMA3C* [29], *DPP4* [30], and *ITGA1* [31], which are reported to enhance cancer cell migration and invasion (Fig. 5d,e). Together, these observations indicate DNAJC10 overexpression alters multiple molecular and cellular processes/pathways in GBM cells, particularly those linked to cell motility. To further confirm if DNAJC10 overexpression alters specific gene set expression, we performed gene set enrichment analysis (GSEA). GSEA results displayed DEGs expressed at lower level in *DNAJC10*-OE group were enriched in the “regulation of glial cell migration” gene set, again supporting *DNAJC10* overexpression suppresses glioma cell migration (Fig. 5f). Notably, GSEA also demonstrated *DNAJC10* overexpression inhibits EGFR signaling pathway (Fig. 5g), in line with KEGG analysis showing *DNAJC10*-OE-induced DEGs are enriched in EGFR downstream pathways (PI3K-Akt, MAPK; Fig. 5c).

### Overexpression of DNAJC10 reduces *EGFR* transcription and inhibits its downstream signaling pathway

**Fig. 6:**
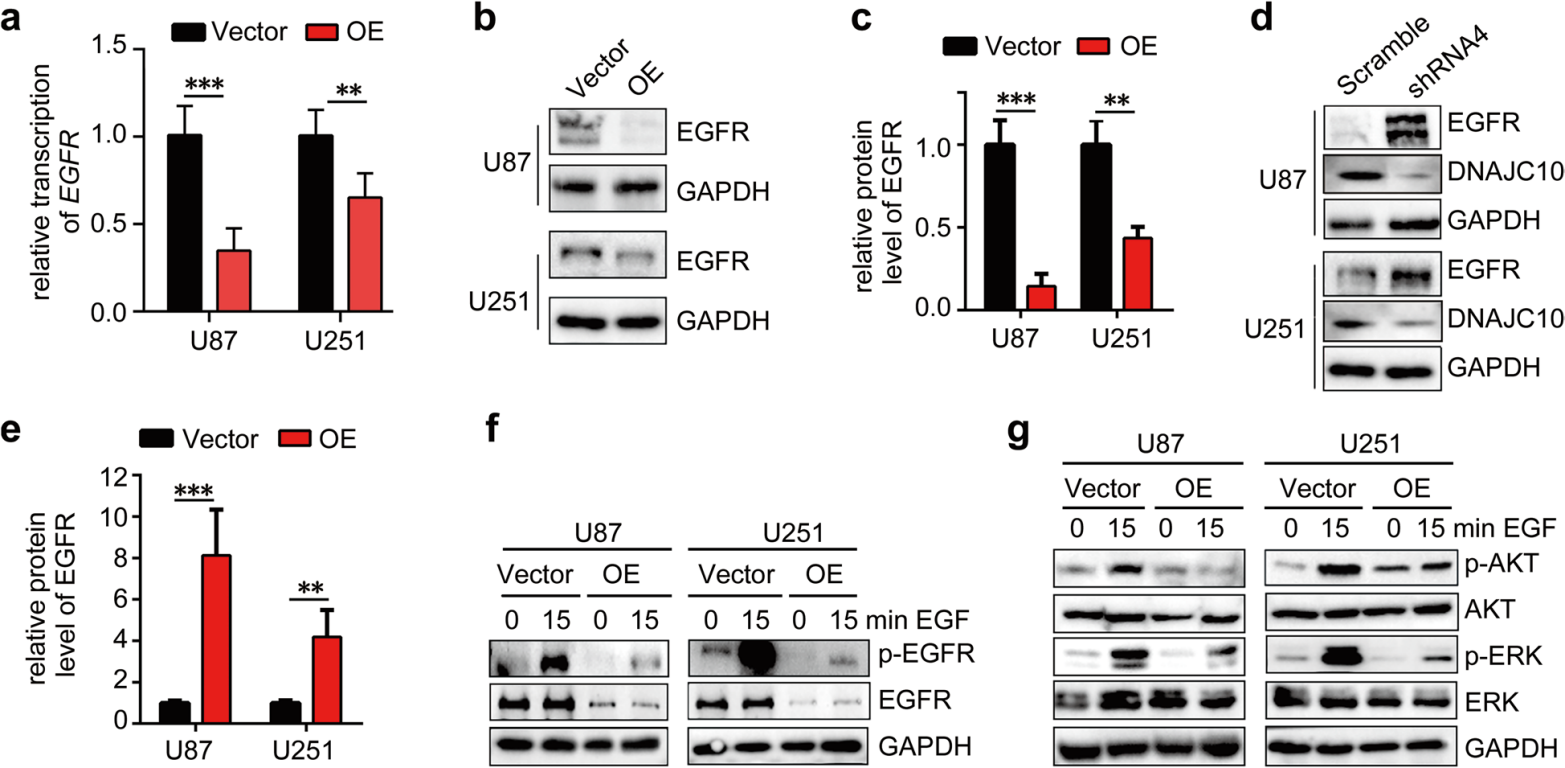
Overexpression of DNAJC10 inhibits EGFR-AKT/ERK signaling via regulating transcription of EGFR. **a** The transcription level of *EGFR* was assessed by RT-qPCR following *DNAJC10* overexpression. (**b-e**) GBM cells were infected with *DNAJC10*-OE (b) or shRNA (d) lentivirus, followed by western blot analysis of expression of EGFR. c, e, Densitometry analysis results for panel b and d was plotted after normalization to GAPDH. (**f, g**) Vector or *DNAJC10*-OE GBM cells were treated with or without EGF, followed by western blot analysis of phosphorylation of EGFR, AKT, and ERK. (All the data are from 3 independent experiments. Multiple t-test, **, *p* < 0.001; ***, *p* < 0.0001.)

RNA-seq results demonstrated DNAJC10 overexpression downregulates EGFR and its downstream molecules. To validate these findings, RT-qPCR was performed to analyze *EGFR* transcription. The results revealed a significant decrease in *EGFR* mRNA level following *DNAJC10* overexpression (Fig. 6a). Consistently, Western blot confirmed EGFR protein expression was significantly downregulated by DNAJC10 overexpression (Fig. 6b,c), conversely, DNAJC10 knockdown increased EGFR expression (Fig. 6d,e).

To assess EGFR activation, we mimicked in vivo conditions by treating cells with EGF and measuring EGFR phosphorylation. Notably, DNAJC10 overexpression significantly reduced EGFR phosphorylation compared to the Vector group (Fig. 6f). Furthermore, we investigated EGFR’s downstream MAPK and PI3K/AKT signaling pathways. AKT and ERK exhibited enhanced phosphorylation levels after adding EGF, however, their phosphorylation decreased upon *DNAJC10* overexpression compared to the Vector group (Fig. 6g). These results suggeste *DNAJC10* overexpression reduces *EGFR* transcription and inhibits its downstream AKT and ERK pathways.

### XBP-1s binds *EGFR* promoter and drives its transcription via histone H3K4 methylation

**Fig. 7:**
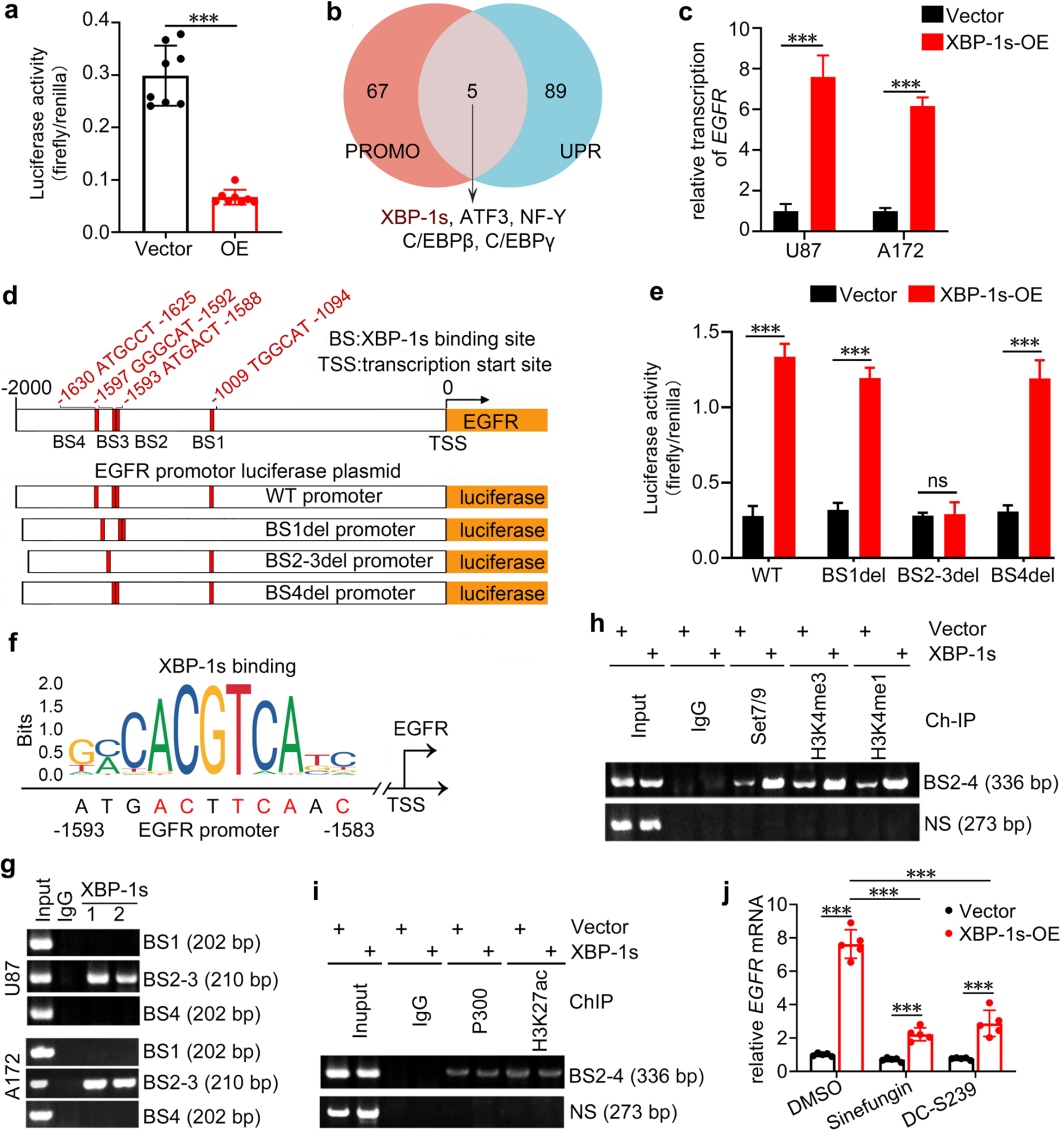
XBP-1s promotes *EGFR* transcription via modifying histone H3K4 methylation. **a** Luciferase activity of *EGFR* promoter was detected in Vector or *DNAJC10*-OE conditions. (**b**) Five candidate transcription factors for *EGFR* promoter were identified through the intersection of the PROMO database and the UPR related genes from PATHCARDS. (**c**) The transcription level of *EGFR* was assessed by RT-qPCR following *XBP-1s* overexpression. (**d**) Schematic outlines of the predicted binding of XBP-1s to *EGFR* promoter (upper panel, data from PROMO) and the design of the WT or mutated *EGFR* promoter luciferase reporter (lower panel). (**e**) Luciferase activity of WT or mutated *EGFR* promoter was detected in Vector or *XBP-1s*-OE conditions. (**f**) Transcription factor binding motif analysis of the XBP-1s binding sites for *EGFR* promoter (data from JASPAR). (**g**) ChIP assays reveal that endogenous XBP-1s specifically binds to the EGFR promoter at the BS2-3 region as opposed to BS1 and BS4. (1 and 2 represent duplicates). (**h, i**) Cells were infected with the Vector or XBP-1s lentivirus, and chromatin fragments were immunoprecipitated with the indicated antibodies. The immunoprecipitated products were subjected to PCR amplification using specific primers, followed by electrophoresis. (NS indicates nonspecific site at −2883 to −2611). (**j**) Vector or XBP-1s-overexpressing cells were treated with the methyltransferase inhibitors Sinefungin or DC-S239. RNA was extracted and EGFR expression was detected by RT-qPCR. (Multiple t-test, ns, no significance; ***, *p* < 0.0001.)

The above findings suggested DNAJC10 regulates *EGFR* transcription. An *EGFR* promoter luciferase assay confirmed this, showing significantly reduced activity in the DNAJC10-OE group versus Vector (Fig. 7a). However, PROMO predictions showed DNAJC10 is not a candidate transcriptional regulator of *EGFR*. Instead, its associated proteins (XBP-1s, ATF3, C/EBPβ, C/EBPγ, NF-Y) were predicted as potential *EGFR*-binding transcription factors, with XBP-1s exhibiting a dissimilarity rate < 5% (Fig. 7b, Table S3). Moreover, XBP-1s overexpression significantly enhanced *EGFR* transcription in U87 and A172 cells (Fig. 7c), supporting that XBP-1s functions as an the upstream transcriptional regulator for the *EGFR* promoter.

Bioinformatics analysis identified four candidate XBP-1s binding sites (BS) in the *EGFR* promoter. Using mutant *EGFR* promoter-driven luciferase reporter plasmids (Fig. 7d, Table S4), dual-Luciferase assays showed XBP-1s upregulation notably increased luciferase activity WT-, BS1del-, or BS4del-*EGFR* promoter sequence, whereas had no effect with BS2-3del-*EGFR* promoter (Fig. 7e), suggesting XBP-1s may directly binds the *EGFR* promoter at BS2-3. Interestingly, sequence alignment showed the conserved XBP-1s binding motif closely matched BS2 (Fig. 7f), further supporting BS2-3 as a critical binding locus.

To validate direct interaction between XBP-1s and *EGFR* promoter, we performed ChIP assays and found endogenous XBP-1s specifically bound the *EGFR* promoter at BS2-3, but not to BS1 or BS4 (Fig. 7g), consistent with the luciferase assays (Fig. 7e). Inspired by literature demonstrating that XBP-1s regulates transcription via histone modifications [32, 33], we explored the mechanism. ChIP assays revealed XBP-1s overexpression significantly enhanced recruitment of SET7/9 methyltransferase and active histone marks H3K4me3 and H3K4me1 at the *EGFR* promoter (Fig. 7h), known biomarkers of transcriptional activation. Conversely, no significant enrichment of acetyltransferase P300 or H3K27ac was detected (Fig. 7i). Functional validation using methyltransferase inhibitors Sinefungin or DC-S239 showed that inhibiting histone methylation attenuated XBP-1s-induced *EGFR* upregulation (Fig. 7j). Together, these data indicate XBP-1s promotes *EGFR* expression primarily by recruiting SET7/9 to induce H3K4 methylation at the promoter, not through acetylation.

### DNAJC10 inhibits XBP-1s by attenuating the IRE1α branch of the UPR

**Fig. 8:**
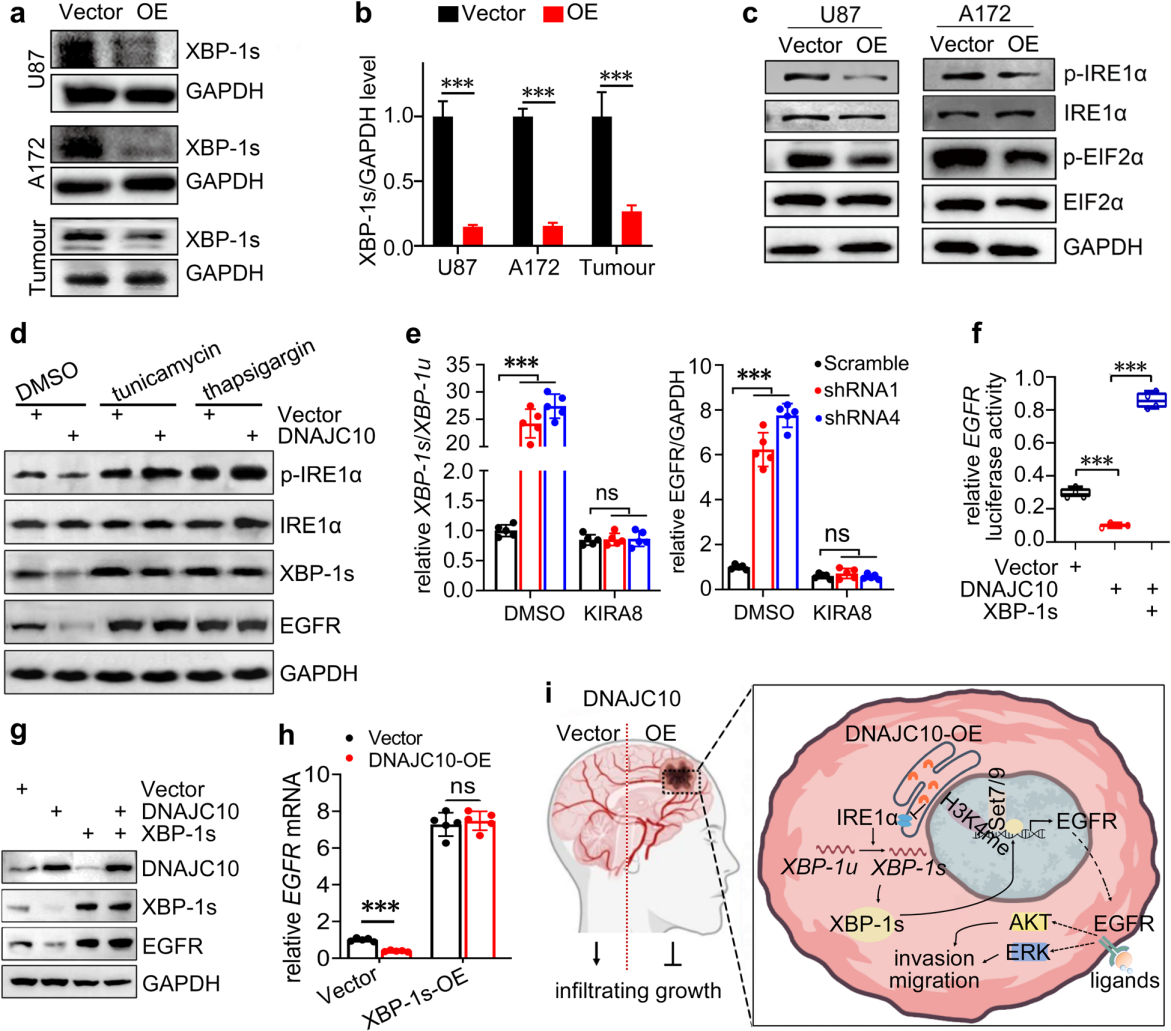
DNAJC10 suppresses XBP-1s by dampening the IRE1α pathway. **a** Western blot analysis of expression of the XBP-1s in Vector or *DNAJC10*-OE GBM cells or in vivo Tumours. (**b**) Densitometry analysis results for panel A was plotted (*N* = 3, Multiple t-test). (**c**) Western blot analysis of activation of the IRE1α and EIF2α in Vector or *DNAJC10*-OE GBM cells. **(d)** Western blot analysis of IRE1α phosphorylation, XBP-1s, and EGFR expression in DNAJC10-OE GBM cells treated with ER stress inducers tunicamycin or thapsigargin. (**e**) RT-qPCR analysis of *XBP-1s* splicing and *EGFR* levels in GBM cells with *DNAJC10* knockdown (shRNA1/shRNA4) and/or IRE1α inhibitor KIRA8 treatment. (*N* = 5, Multiple t-test) (**f**) Luciferase activity of *EGFR* promoter was detected in the indicated conditions (Student’s t-test). (**g**) Western blot analysis of expression of XBP-1s and EGFR in the indicated conditions. (**h**) RT-qPCR analysis of *EGFR* transcription in the indicated conditions (*N* = 5, Multiple t-test). **(i)** Proposed working model of how DNAJC10 regulates invasion and migration of GBM cells. (, ***, *p* < 0.0001; ns, no significance)

Having established XBP-1s drives *EGFR* transcription, we next investigated DNAJC10-mediated XBP-1s regulation. Western blot revealed DNAJC10 overexpression significantly reduced XBP-1s protein levels in GBM cells and in vivo tumours (Fig. 8a,b). Since XBP-1s activation requires ER stress-induced splicing via UPR, we examined UPR components. DNAJC10 overexpression suppressed phosphorylation of eIF2α (Fig. 8c), consistent with its known role in attenuating [20].

Concurrently, DNAJC10 overexpression also inhibited IRE1α phosphorylation (Fig. 8c,d), critical for XBP-1 splicing. Pharmacological rescue with ER stress inducers thapsigargin and tunicamycin partially restored IRE1α phosphorylation, XBP-1s expression, and EGFR levels in DNAJC10-overexpressing cells (Fig. 8d), linking DNAJC10 to IRE1α/XBP-1s suppression. Conversely, DNAJC10 knockdown enhanced XBP-1s splicing and *EGFR* transcription (Fig. 8e), effects abrogated by IRE1α inhibitor KIRA8 (Fig. 8e). This confirms the essential role of the IRE1α branch in DNAJC10-mediated XBP-1s regulation. To determine if XBP-1s is responsible for DNAJC10-mediated *EGFR* transcription supression, Luciferase assays showed DNAJC10-induced repression of *EGFR* promoter activity was rescued by XBP-1s overexpression (Fig. 8f). Moreover, XBP-1s overexpression reversed DNAJC10-induced EGFR downregulation at protein and transcriptional levels (Fig. 8g,h). Collectively, DNAJC10 inhibits *EGFR* transcription by suppressing XBP-1s activation through attenuation UPR IRE1α branch.

## Discussion

DNAJC10 is an ER-resident reductase that cleaves disulfide bonds of misfolded proteins [16]. Emerging evidence implicates DNAJC10 in cancer processes including apoptosis, proliferation, autophagy, migration, and invasion, though its role in GBM remains poorly defined. We proposed DNAJC10 as a tumor suppressor based on its downregulation correlating with poor patient outcomes, supression of migration and invasion, and inhibition of tumor propagation in xenografts. This aligns with DNAJC10’s tumor-suppressive roles in prostate [19], neuroblastoma [20], and colon cancer [21] via modulating UPR. Specifically, DNAJC10 inhibits the PERK-eIF2α pathway, disrupting survival mechanisms and promoting apoptosis. However, DNAJC10 also promotes leukemia stem cell self-renewal during ER stress [18], and its knockdown reduces proliferation in lung adenocarcinoma [17], indicating its context-dependent oncogenic roles.

These divergent pro-oncogenic and tumor-suppressive roles may arise from lineage-specific ER stress dependencies. Leukemia stem cells in chronically stressed microenvironments (e.g., hypoxia) experience UPR-mediated overactivation of the PERK-eIF2α pathway, triggering apoptosis. These cells rely on DNAJC10 to dampen this overactivation and prevent apoptosis [18]. Conversely, neuroblastoma and prostate cancer cells with low ER stress tolerance depend on mild PERK-eIF2α activation to survive, reducing protein synthesis [20]. DNAJC10 blocks this adaptation, inducing apoptosis in these cells. This highlights cell-type-specific ER stress thresholds and UPR pathway biases. Furthermore, our findings suggest that the dual roles of DNAJC10 may be attributed to its ability to regulate a wide range of proteins or pathways depending on the cell type/lineage or specific stimulus [12]. Hence, further studies are needed to explore the role of DNAJC10 in cancer as a ‘‘friend or foe’’ and to elucidate its clinical significance in difference cancers.

While EGFR amplification and XBP-1 dysregulation are established GBM hallmarks [34–36], their upstream regulation and crosstalk are incompletely understood. We identify a novel DNAJC10–XBP-1s–EGFR axis linking ER stress to *EGFR* transcriptional control. DNAJC10 suppresses *EGFR* transcription by inhibiting XBP-1s activation through the UPR IRE1α branch, distinct from known EGFR regulators. Crucially, XBP-1s acts as a direct EGFR transcriptional activator: bioinformatics, dual-luciferase assays, and ChIP confirm it binds the EGFR promoter promoter (BS2–3 region), recruits SET7/9 methyltransferase, and induces H3K4me3/me1 histone marks. Further, Histone methylation inhibition abrogates XBP-1s-mediated *EGFR* upregulation. Clinically, DNAJC10 downregulation correlates with poor GBM survival, highlighting its tumor-suppressive role by disrupting XBP-1s–EGFR axis. Targeting DNAJC10–XBP-1s–EGFR axis offers a rationale for combinatorial therapies against EGFR-resistant GBM.

Notably, our findings hold clinical significance as GBM infiltration impedes surgical resection. DNAJC10 overexpression intracranial tumors exhibited attenuated invasion and defined boundaries, suggesting DNAJC10 might to be a novel anti-invasive target. Fortunately, brain-targeted nanoparticles could facilitate DNAJC10 delivery, though clinical benefits require further study.[37, 38]. Importantly, RNA-seq indicated DNAJC10 downregulates CAMs-related genes such as ITGAV [28], SEMA3C [29], DPP4 [30], and ITGA1 [31], which promote cancer cell migration and invasion. Given the complexity of overexpression DNAJC10 in GBM patients, molecular inhibitors of these genes may offer alternative therapeutic strategies.

RNA-seq also revealed DNAJC10’s involvement in cancer-related pathways, including but not limited to focal adhesion, ECM-receptor interaction, PI3K-Akt signaling, regulation of actin cytoskeleton, and MAPK signaling pathway. GSEA suggested DNAJC10 suppresses EGFR signaling, consistent with EGFR’s involvement in these pathways. Accordingly, we hypothesized that EGFR may be one of the most important target genes of DNAJC10 regulating infiltration and metastasis of GBM cells. We confirmed DNAJC10 overexpression downregulates EGFR mRNA/protein and attenuates AKT/ERK activation, suggesting the EGFR-AKT/ERK axis mediates DNAJC10’s effects in GBM. While focused on EGFR, DNAJC10-dependent downregulation of PERK-ATF4 and IRE1α-JNK targets implies broader regulatory roles warranting future investigation.

*EGFR* transcriptional regulation is complicated and remains largely unknown. While TIEG1[39] and Y-box binding protein 1 (YB1) [40] directly bind the *EGFR* promoter to regulate cancer progression, we demonstrate XBP-1s binds the *EGFR* promoter. Beyond its role in UPR target genes [41, 42], XBP-1s also regulates transcription of aminopeptidase *TPP2* [43] and Δ9 desaturase *FAT-6* [44], which is a key enzyme in fatty acid metabolism regulation. Here, we extend this paradigm by demonstrating XBP-1s binds the −1597 to −1588 region at *EGFR* promoter. Mechanistically, XBP-1s recruits SET7/9 methyltransferase to induce H3K4me3/me1 marks at the *EGFR* promoter. Pharmacological inhibition of histone methylation blocks XBP-1s-mediated *EGFR* upregulation, establishes a novel epigenetic mechanism distinct from its canonical UPR functions. Future ChIP-seq studies could determine if DNAJC10 broadly restrains XBP-1s binding and H3K4 methylation genome-wide, thereby strengthening the causal chain of this regulatory axis. Nonetheless, these findings elucidate DNAJC10/XBP-1s-induced epigenetic modification of EGFR and suggest therapeutic implications for EGFR inhibition.

EGFR signaling-targeted agents (e.g., monoclonal antibodies, small molecule tyrosine kinase inhibitors) are advanced in clinical development [45]. Yet, EGFRvIII – present in ~ 30% of GBM patients – undermines their efficacy in gliomas [24, 46]. Although novel anti- EGFR antibodies, such as monoclonal antibody 806, can target EGFRvIII, understanding the transcriptional regulatory mechanisms of *EGFR* promoter is still important. Crucially, XBP-1s overexpression upregulated *EGFR* transcription in both wild-type U87 and EGFRvIII-expressing A172 cells [47]. Thus, targeting the transcriptional regulator for *EGFR* promoter, such as XBP-1s, may be a novel strategy to overcome EGFR inhibitor resistance. DNAJC10-XBP-1s’ suppression of *EGFR* transcription provides a mechanistic basis for disrupting EGFR-dependent survival pathways in resistant GBM.

The study has several limitations that warrant discussion. Firstly, although survival analysis was strengthened by integrating TCGA and CPTAC datasets, the clinical sample size for the IHC analysis was small, and the correlation between mRNA and protein expression of DNAJC10 was inconsistent [48], suggesting post-translational complexity or immunological factors. Secondly, the research primarily relies on in vitro experiments and xenograft models, lacking validation in patient-derived organoids or genetically engineered mouse models for more clinically relevant assessment of DNAJC10’s anti-invasive effects. Additionally, while DNAJC10’s functions were validated across multiple cell lines, its expression heterogeneity and differential roles in distinct GBM subtypes (such as classical and mesenchymal subtypes) remain uncharacterized, potentially limiting generalizability. Furthermore, although DNAJC10 regulates *EGFR* transcription via the IRE1α/XBP-1s axis, crosstalk with other UPR branches (such as PERK/eIF2α, IRE1α-JNK) and its microenvironmental effects (such as immune cell infiltration) require deeper exploration. Lastly, the potential long-term toxicity of DNAJC10 overexpression in normal brain tissues requires further in vivo verification for translational safety.

## Conclusions

In conclusion, DNAJC10 inhibits GBM migration, invasion, and tumor propagation in vitro and in vivo by targeting EGFR. Mechanistically, we revealed a novel DNAJC10–XBP-1s–EGFR axis. DNAJC10 suppresses UPR IRE1α branch and reduces XBP-1s activation. XBP-1s directly binds the *EGFR* promoter and recruits SET7/9 methyltransferase to induce H3K4 methylation, a mechanism distinct from known EGFR regulators (Fig. 8i). This expands understanding of *EGFR* transcriptional control and UPR-oncogenic crosstalk. Clinically, DNAJC10 downregulation correlates with poor survival, while targeting the DNAJC10/XBP-1s/EGFR axis offers a novel therapeutic strategy for EGFR inhibitor-resistant GBM. Importantly, in vitro and in vivo [49] experiments show minimal impact of DNAJC10 overexpression on normal glial cells, supporting its therapeutic safety. Collectively, these results establish s–EGFR axis as critical in GBM infiltration and propose DNAJC10 as a promising biomarker and therapeutic candidate for overcoming treatment resistance.

## Materials and methods

### Regents and antibodies

D-Luciferin (ab143655) used for in vivo imaging is purchased from Abcam. Thapsigargin (HY-13433), tunicamycin (HY-A0098), KIRA8(HY-114368), Sinefungin (HY-101938), and DC-S239 (HY-121093) is purchased from MedChemExpress. Rhodamine-labeled Phalloidin used for cytoskeletal staining is purchase from Solarbio (No.CA1610). Antibodies used for immunohistochemistry (IHC) or Western blot (WB) or ChIP are as follows: DNAJC10 (13,101–1-AP), EGFR (66,455–1-IG), AKT (10,176–2-AP), GRP78 (11,587–1-AP), P-AKT S473 (66,444–1-Ig), and GAPDH (10,494–1-AP) are from Proteintech. P-EGFR Y1068 (3777), XBP-1s (40,435), N-Cadherin (13,116), Anti-ZO-1 (13,663), Vinmentin (5741), EIF2α (5324), P-EIF2α S51 (3597), H3K4me3(9751), H3K4me1(5326), P300 (54,062) and H3K27ac (8173) are derived from Cell Signaling Technology. Anti-ERK1/2 (44W4852) and Anti-P-ERK1/2 (87B4599) are derived from Affinity.

### Cell culture

The GBM cell lines U87 (EGFR wild type) (#HTB-14), U118 (HTB-15), and A172 (EGFRvIII mutation) (CRL-1620), and HEK-293 T (CRL-3216) cells were obtained from the American Type Culture Collection (ATCC). U251 (BFN608006387), and normal human astrocytes HA1800 (BFN60804059) cells were obtained from Cell Lines Bank, Chinese Academy of Science (Shanghai, China). All the cell lines were characterized by DNA fingerprinting and isozyme detection and tested to be mycoplasma-free and were cultured with DMEM (sigma) supplemented with 10% fetal bovine serum (Gibco) in a controlled environment at 37℃ with 5% CO_2_.

### Immunohistochemistry (IHC)

Tumor specimens (including human clinical CRC samples and xenografted tumors) were fixed and paraffin-embedded. Five-micrometer tissue sections were prepared using a rotary microtome. IHC staining was performed with the labeled streptavidin–biotin detection system according to the manufacturer’s instructions. A semi-quantitative scoring system was applied by two independent pathologists blinded to clinical data. Staining intensity was graded as: 0 (negative), 1 (weak), 2 (moderate), or 3 (strong). The percentage of positive tumor cells was categorized as: 0 (≤ 5%), 1 (6–25%), 2 (26–50%), 3 (51–75%), or 4 (≥ 76%). The final immunoreactivity score (IRS) was calculated by multiplying intensity and percentage scores, with a theoretical range of 0–12. Quantitative analysis used ImageJ software with the IHC Profiler plugin to calculate integrated optical density values. Five representative fields per section were analyzed via systematic random sampling.

### Plasmid and Lentivirus construction and infection

shRNAs targeting human DNAJC10 or a Scrambled shRNA were cloned into PLL3.7 lentiviral vector. The targeted sequences are shown in Table S1. The lentiviral Vector plasmid and DNAJC10 or XBP-1s overexpression plasmid pSLenti-CMV-DNAJC10 and pSLenti-CMV-XBP-1s was obtained from OBiO Technology (Shanghai, China). Lentivirus production and cell infection was performed as our previously report [20]. Briefly, the lentivirus constructs were mixed with pSPAX2 and pMD2.G (4:3:1), followed by transfection into HeK- 293 T cells using polyJet. The lentivirus-containing supernatant was collected at 48 and 72 h post-transfection. For infection, cells were maintained in lentivirus supernatant supplemented with 4 µg/ml polybrene and centrifuged at 1800 rpm for 2 h at 37 ◦C. After continued cultured in lentiviral supernatant for 4 h, the lentivirus supernatant was discarded and replaced with DMEM medium with 10% FBS. 24 hours later, the procedures were repeated for the secondary infection. Cells carrying EGFP or RFP in the plasmid can be sorted using flow cytometry.

### Cell migration and invasion assay

The Trans-well cell assay was divided into two types: substrate gel-coated and non- coated cells, respectively, to assess the invasive and migratory capabilities of the cells. Firstly, the cells were starved for 24 h in the FBS-free medium. Then the starved cells were seeded in the upper chamber of the Trans-well inserts (1 × 10^5^ cell/well, N = 4), while the lower chamber contained DMEM supplemented with 20% FBS. The chambers were then incubated at 37℃ for 24 h in matrix glue-coated wells and for 10 h in non-glue coated wells. Then, the cells beneath the upper chambers were fixed with 4% paraformaldehyde, stained with crystal violet. Finally, images from five different areas were captured for each well. Cell wound scratch assays were performed as we previously described [50].

### Quantitative real-time PCR

Total RNA was extracted by Trizol method, reverse transcribed with PrimeScript RT Enzyme Mix I (Takara) reverse transcriptase primers, and quantitative real-time PCR (RT-qPCR) was performed using SYBR Green PCR Mix (Applied Biosystems) and IQ5 detection system (Bio-Rad, Hercules, CA). The primer sequences are shown in Table S1.

### *In vivo *animal studies

The animal studies has been approved by the Animal Ethics Committee of Binzhou Medical University (No. 2021–122). NSG mice aged 4 to 6 weeks were purchased from Shanghai Model Organisms and maintained in a specific pathogen-free (SPF) environment. Sample sizes were minimized following the 3R principles (Replacement, Reduction, Refinement) while maintaining statistical rigor (cohen’s d ≥ 1.38, Power ≥ 85%). To compare the invasion and infiltration abilities in vivo between the Vector control cells (labeled with GFP) and the *DNAJC10*-OE cells (labeled with RFP), dual-fluorescence assay was performed. To eliminate confounding effects of differential GBM cell growth on interpreting invasion/migration, a 1:1.5 mixture of GFP-labeled Vector cells and RFP-labeled *DNAJC10*-OE cells were stereotactically injected into mouse brains (2 mm lateral, 1 mm posterior to the bregma, and 3.0 mm deep). At 28 days post-injection, the mice were subjected to cardiac perfusion with 4% paraformaldehyde (PFA) in PBS. The Stripped brain tissue were frozen in Liquid nitrogen and fixed again with 4% PFA. Then the brain sections were subjected to frozen slices with the Cryostat. Confocal microscope was used to examine the slices of the brain sections. To test the development of GBM in vivo, Vector or *DNAJC10*-OE luciferase constitutive expressed U87 cells were injected into the right caudate nucleus/putamen (1 × 10^6^ cells/mouse in 10 μL PBS). After the orthotopic xenograft models were established, the mice were separately utilized for bioluminescence imaging, Magnetic Resonance Imaging (MRI), or the calculation of overall as we previously reported [51]. The brain tissues from the MRI group were subjected to H&E staining and histological analysis.

### RNA-seq and data analysis

RNA-seq and data analysis was performed as our previous report [51]. Briefly, total RNA was extracted from Vector or *DNAJC10*-OE U87 cells and was submitted to the BGI (Beijing Genomics Institute) for RNA sequencing. The candidate genes with |fold change|≥ 2 and adjusted *p*-value < 0.05 were defined as DEGs. The DEGs were subjected to Heatmaps analysis, gene ontology (GO) enrichment, Kyoto Encyclopedia of Genes and Genomes (KEGG) enrichment, and gene set enrichment analysis (GSEA), respectively.

### Dual-Luciferase reporter assay

The wild type (WT) or mutated promoter of *EGFR* was cloned into the pGL3 luciferase vector (Promega Corporation). The *EGFR* promoter-driven luciferase reporter plasmid along with Vector, *DNAJC10*-OE, or *XBP-1s*-OE plasmid was transfected into HeK- 293 T cells following the instructions of the manufacturer. At 48 h post-transfection, cells were harvested and probed for luciferase in the Dual Luciferase® Reporter Assay System. Renilla Luciferase activity was used as internal control.

### Chromatin Immunoprecipitation (ChIP) Assay

The ChIP assay was performed to analyze protein-DNA interactions as described previously [52]. U87 or A172 cells were cross-linked with 1.4% formaldehyde for 15 min at room temperature, quenched with 125 mM glycine, and lysed in IP buffer containing protease/phosphatase inhibitors. Chromatin was sheared by sonication to obtain 200–1,000 bp fragments. Lysates were precleared and incubated with IgG or indicated antibodies (2 μg/IP) in an ultrasonic bath at 4 °C for 15 min. Protein A-Sepharose beads were added and rotated at 4 °C for 45 min to capture immune complexes. Beads were washed 5–6 times with IP buffer, and DNA was isolated using Chelex 100: beads were boiled for 10 min, treated with proteinase K (20 μg/μl) at 55 °C for 30 min, and boiled again to inactivate enzymes. Supernatants were collected and used for PCR. Primers used for ChIP assays are described in Table S1.

### Statistical analysis

The experimental data were analyzed using IBM SPSS 23.0 software, and statistical charts were generated using GraphPad Prism. All data were represented as mean values ± standard deviations. A Student’s t-test was employed to compare the difference between the two groups, while a multiple t-test analysis of variance was performed on the data above the two groups. A significance level of P < 0.05 was used to indicate that the data exhibited statistical significance.

## Supplementary Information

Supplementary Material 1.

## Data Availability

Data are available from NCBI database (Accession number: PRJNA1306552).
