# Supplementary material for: Endoplasmic reticulum-resident protein DNAJC10 inhibits glioblastoma metastasis by suppressing XBP-1s-driven EGFR transcription
Source: Mol Biomed. 2025 Oct 24;6:84. doi: 10.1186/s43556-025-00308-0 (PMC12589755; doi:10.1186/s43556-025-00308-0)
Supplement: Supplementary file 1 — Supplementary Material 1. [file 43556_2025_308_MOESM1_ESM.docx]

**Endoplasmic reticulum-resident protein DNAJC10 inhibits glioblastoma metastasis by suppressing XBP-1s-driven EGFR transcription**

Erdi Zhao^1,†^, Yue Yu^2,†^, Yingli Gao^1,†^, Teng, Li^1,†^, Shiyu Hao^1^, Meiyang Chen^1^, Ming Xu^1^, Sinkemani Arjun^1^, Chunyan Yang^3,4^, Yancun Yin^1,*^, Minjing Li^1,5*^

^1^ Laboratory of Experimental Hematology, School of Basic Medical Sciences, Binzhou Medical University, Yantai 264003, China.

^2^ The Second School of Clinical Medicine, Binzhou Medical University, Yantai, 264003, China.

^3^ Institute of Stomatology, Binzhou Medical University, Yantai 264003, China.

^4^ The affiliated Yantai Stomatological Hospital, Binzhou Medical University, Yantai 264003, China.

^5^ School of Traditional Chinese Medicine, Binzhou Medical University, Yantai 264003, China.

**^†^ These authors contributed equally to this work.**

* Corresponding authors

Minjing Li, E-mail: liminjing512@126.com; School of Traditional Chinese Medicine, Binzhou Medical University, Yantai 264003, China. Phone：+86-15553572836.

Yancun Yin, E-mail: yinyc1985@126.com; Laboratory of Experimental Hematology, School of Basic Medical Sciences, Binzhou Medical University, Yantai 264003, China.

Phone：+86-15589558736; Fax: +86-0535-6913213.

**Running title:** DNAJC10 inhibits metastasis of glioblastoma

**Competing interests**

The authors report no competing interests.

**Supplementary Figures and tables**

**
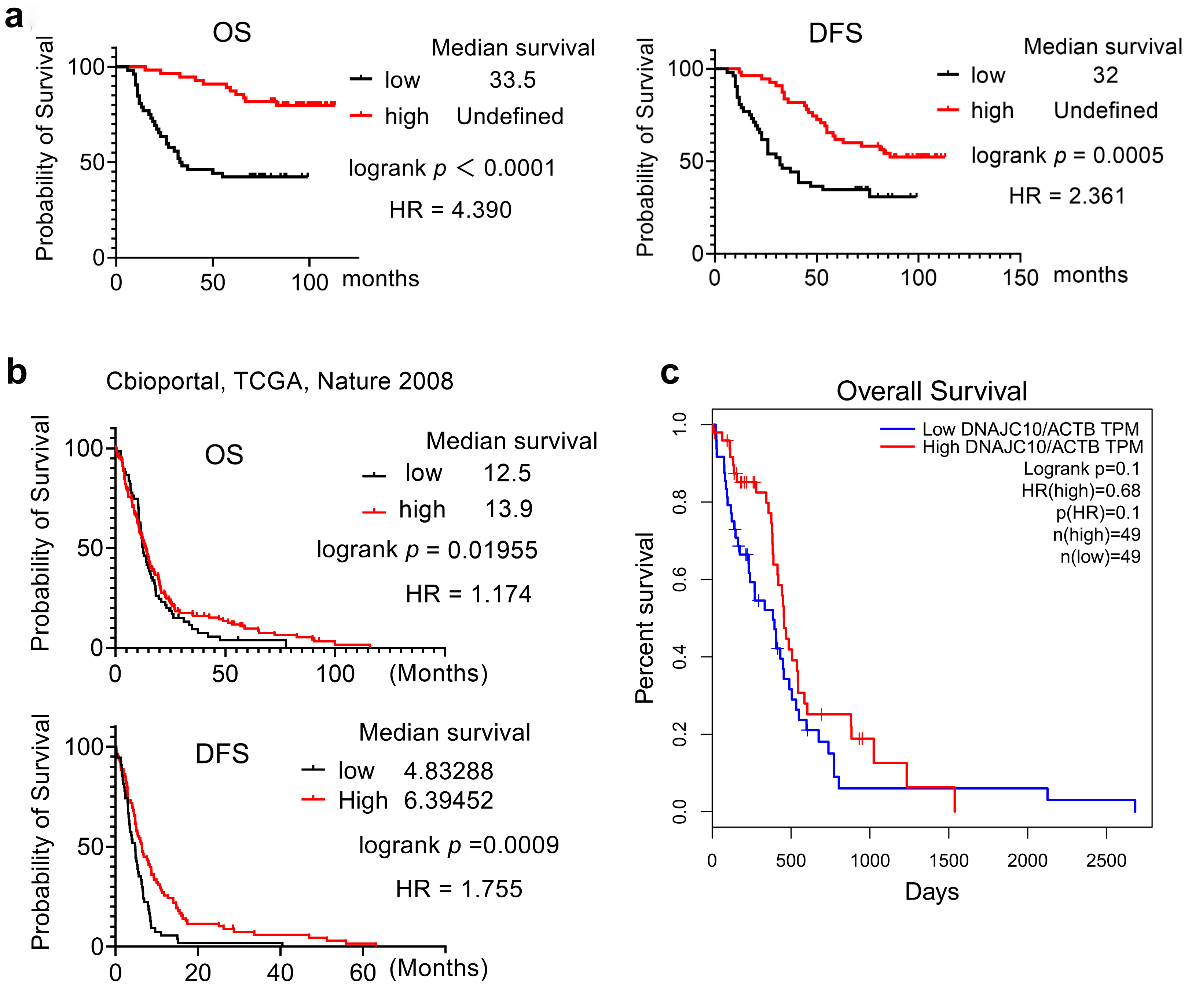
**

Sup. Fig.S1. low expression of *DNAJC10* correlates with the poor overall survival in GBM patients. a, Kaplan-Meier survival curves of patients with low or high DNAJC10 expression (GBM microarray: #HBraG177Su01). b, Kaplan-Meier analysis of survival of GBM patients relative to *DNAJC10* mRNA expression levels in the CbioPortal, TCGA database. c, Kaplan-Meier analysis of overall survival (OS) of GBM patients relative to *DNAJC10* mRNA expression levels in the GEPIA.


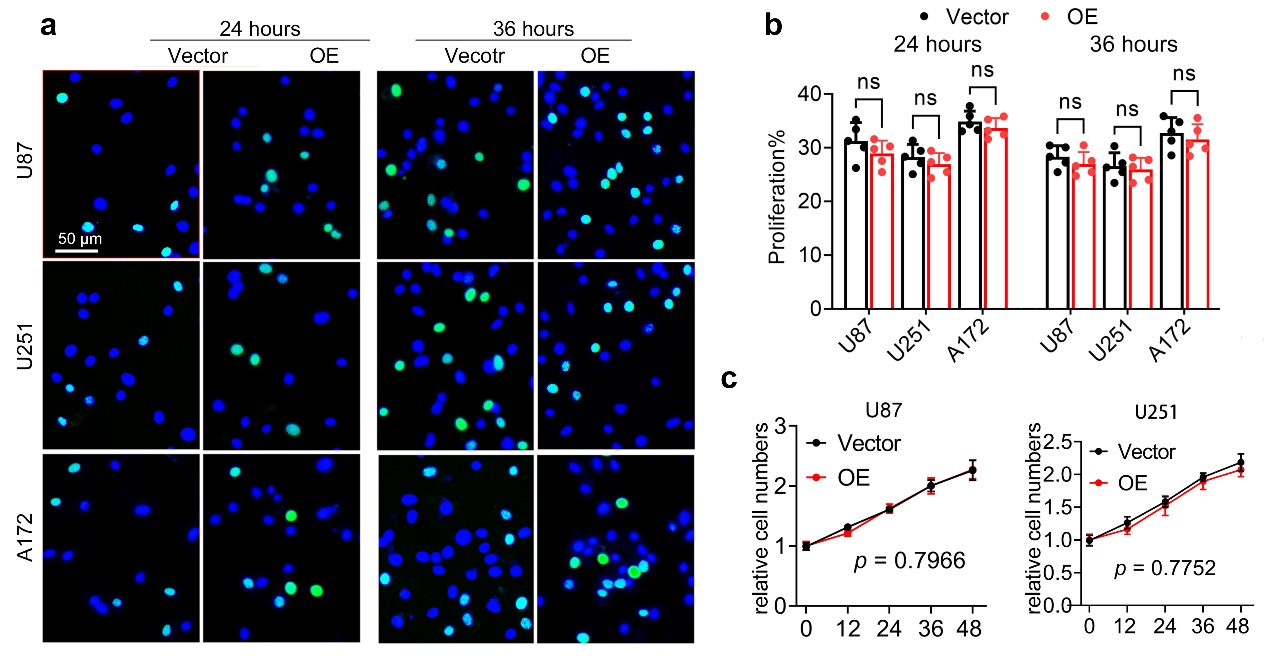


Sup. Fig.S2. Overexpression of DNAJC10 does not affect the proliferation of GBM cells. a-**b**, The effect of DNAJC10 on GBM cell proliferation was determined by EdU labeling. The representative images (a) and proliferation rate (b) was plotted. n = 5. ns, no significant. **c,** relative cell numbers at indicated days after planting. n = 5.

**
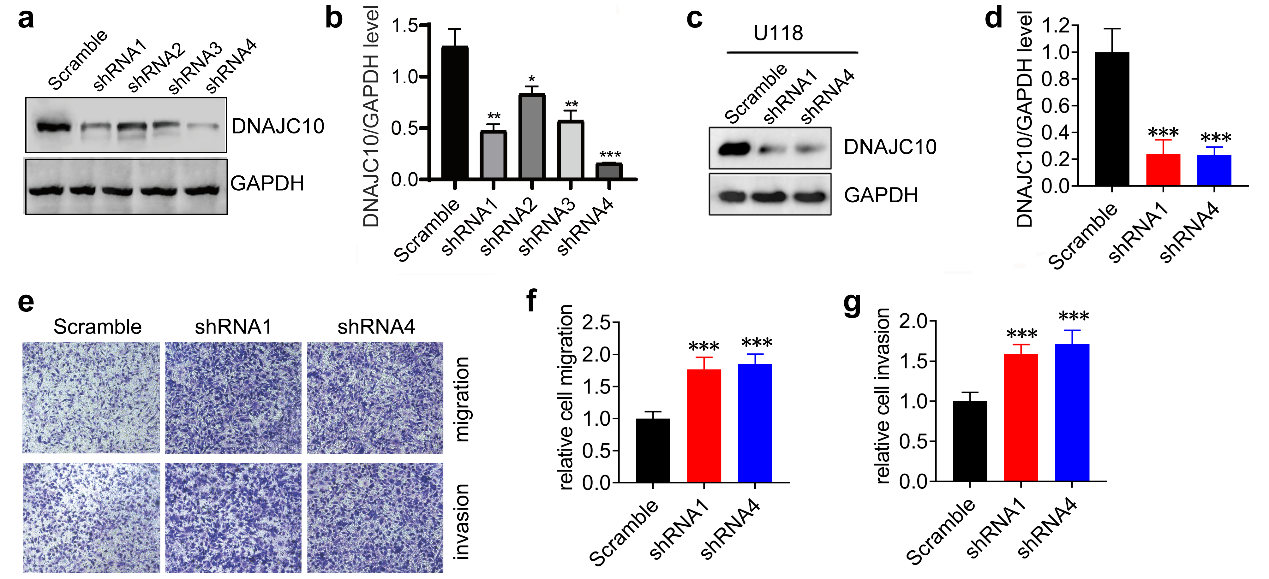
**Sup. Fig.S3. **Knockdown of DNAJC10 promotes migration and invasion of GBM cells.** **a,** Western blot analysis the expression of DNAJC10 in 293T cells transfected with Scramble or the indicated *DNAJC10* shRNA. **b,** The knock-down efficiency of the indicated shRNAs was analyzed. shRNA4 with the highest knockdown efficiency was used for next assays. **c,** Western blot showed that DNAJC10 knockdown U118 cell lines were constructed by lentivirus infection. **d,** Relative expression of DNAJC10 was analyzed in panel C. **e-g,** Trans-well assays were performed to compare migration and invasion ability in the Scramble or shRNA lentivirus infected cells. Representative images of cell migration (f) and cell invasion (g).

**
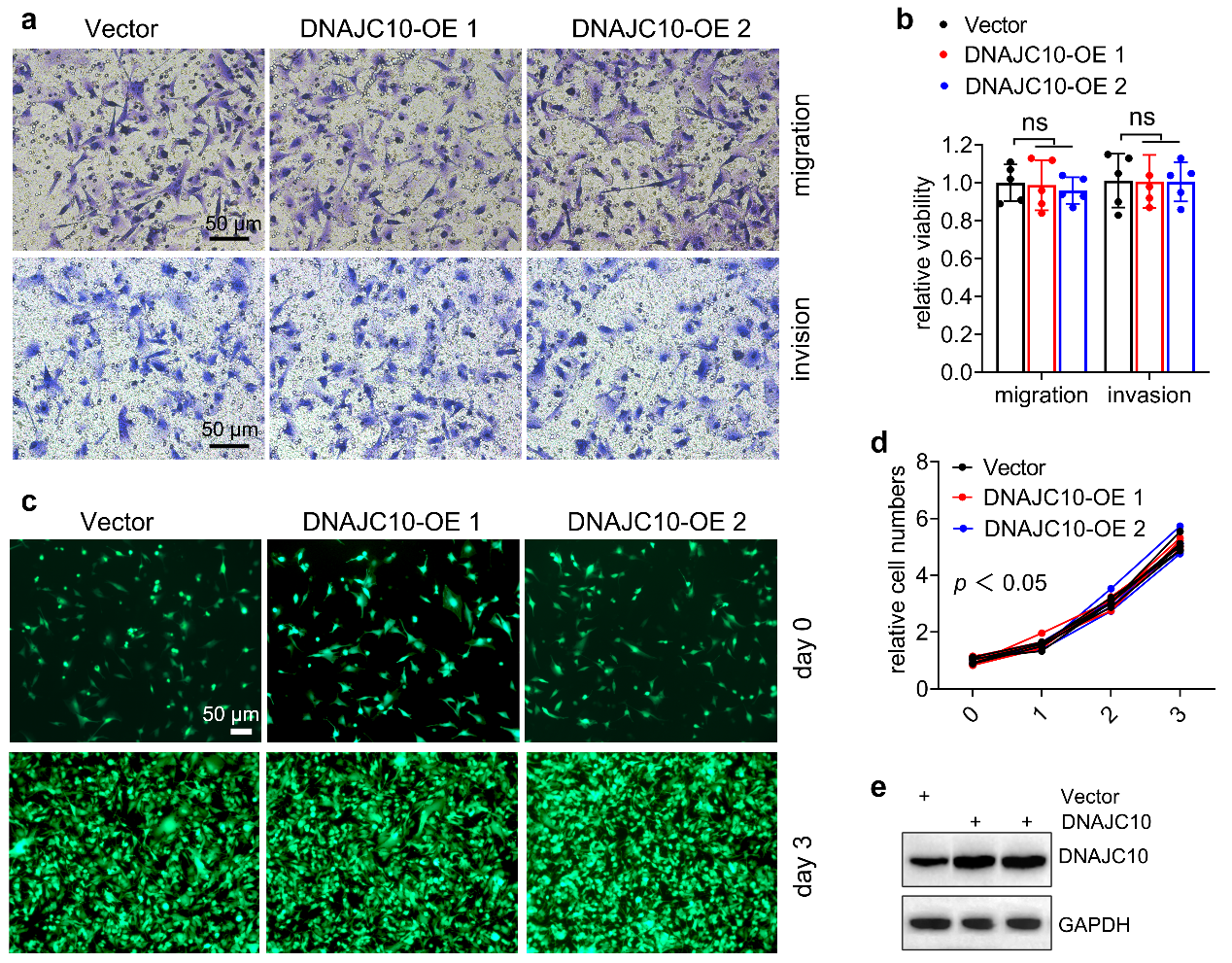
Sup. Fig. S4.** Overexpression of DNAJC10 does not affect the function of normal glial cells. a,Trans-well assays were performed to compare migration (chamber without matrix glue) and invasion (chamber coated with matrix glue) ability in the Vector or DNAJC10-OE lentivirus infected HA1800 cells. Representative images of cell migration and cell invasion. b, relative migrated cells and invaded cells per field. c, Representative images of Vector or DNAJC10 infected HA1800 cells. d, relative cell numbers at indicated days after planting. n = 5. e, Western blot analysis expression of DNAJC10 in the HA1800 cells infected with Vector or DNAJC10-OE lentivirus.


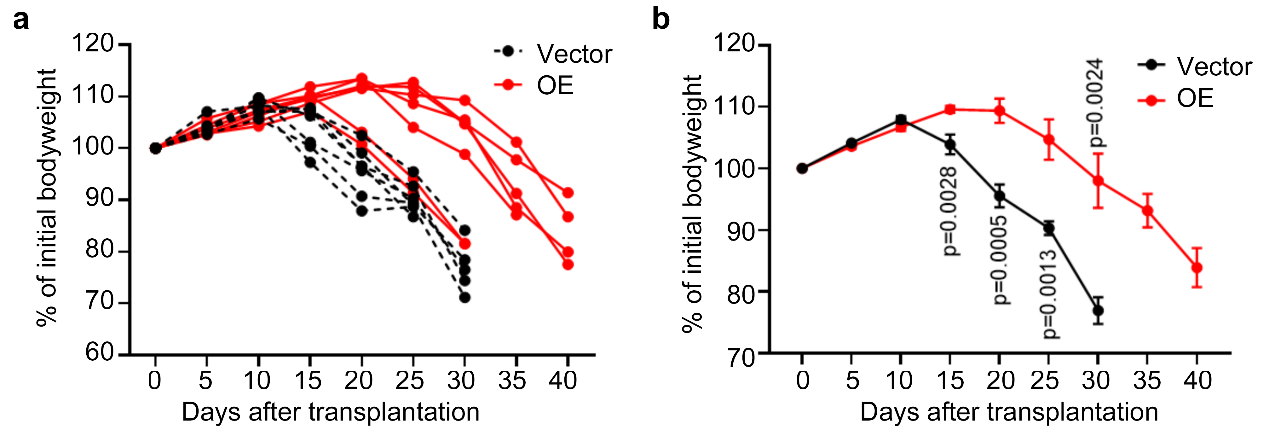


**Sup. Fig. S5.** Body weight of the xenografted mouse. DNAJC10-OE U87 cells were intracranially injected into NSG mice aged 6-8 weeks. Bodyweight of each mouse was monitored every five days until the mouse dead. a, bodyweight loss for the each xenografted mouse. b, the average bodyweight loss of the mice in Vector or OE group (N = 7, maen ± SEM).


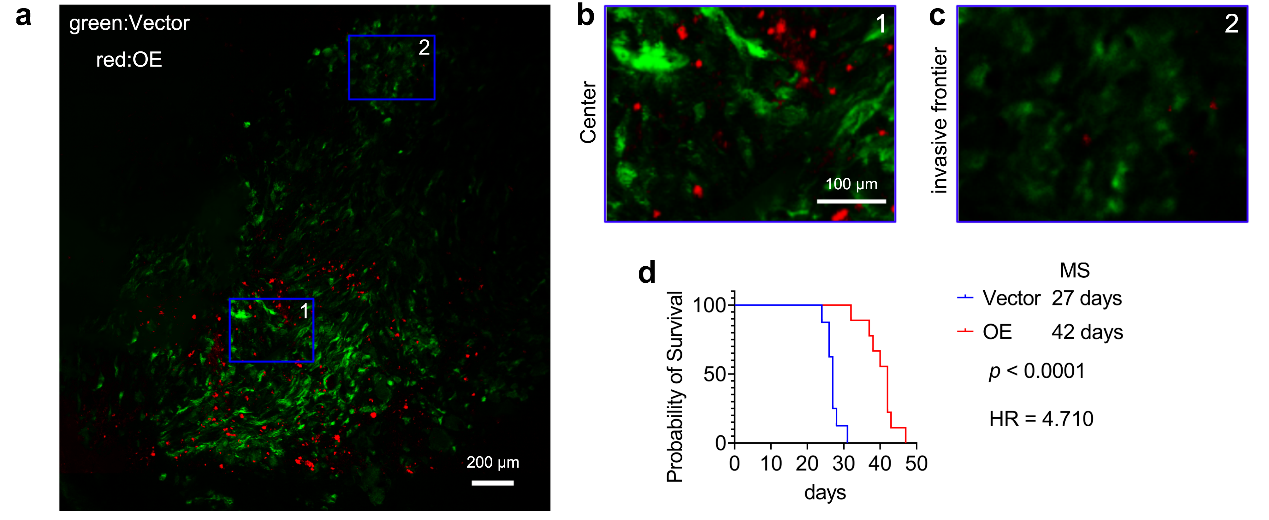


**Sup. Fig. S6.** Overexpression of DNAJC10 in A172 cells inhibits GBM cells invasion in vivo. **a**, A mixture of GFP- labeled Vector cells and RFP-labeled DNAJC10-OE A172 cells (Vector:OE = 1:1.5) was injected into the NSG mouse brain. The brain sections of the xenografted mouse was subjected to frozen section and dual-fluorescence at 30 days post-transplantation. Images showed the (**b**) center (**c**) and periphery of the xenografted tumor. **d,** OS of the xenografted mice was analyzed by Kaplan-Meier and log-rank test (Vector N=8; OE N=9).

| **Gene name** | **Primer/Target Sequence（5’---3’）** |
| --- | --- |
| **shRNA for human DNAJC10** | **Target sequence** |
| Scramble | GATATGTGCGTACCTAGCAT |
| shRNA 1 | GCCGAATGAAAGGAGTCAA |
| shRNA 2 | GCCCCAGTGAAATATCATG |
| shRNA 3 | GCATTGAAGTTACATCCTGAT |
| shRNA 4 | GCACCAGACATCTGTAGTAAT |
| **Primer for PCR** | **Primer** |
| Human *EGFR*-F | AGGCACGAGTAACAAGCTCAC |
| Human *EGFR*-R | ATGAGGACATAACCAGCCACC |
| Human *GAPDH*-F | GGAGCGAGATCCCTCCAAAAT |
| Human *GAPDH*-R | GGCTGTTGTCATACTTCTCATGG |
| Human *XBP-1S*-F | TCTGCTGAGTCCGCAGCAG |
| Human *XBP-1S*-R | GAAAAGGGAGGCTGGTAAGGAAC |
| Human *XBP-1U*-F | TGGTTGCTGAAGAGGAGGCGGAAG |
| Human *XBP-1U*-R | GAGATGTTCTGGAGGGGTGACAACTG |
| **Primer for ChIP PCR** | **Primer** |
| *EGFR*-promotor BS1-F | TAGCTCAAGTTCCTGCAGCC |
| *EGFR*-promotor BS1-R | TGAAGCCAATGTGTGAAGCA |
| *EGFR*-promotor BS2-3-F | CCTTCATGGCCTCTGCATTC |
| *EGFR*-promotor BS2-3-R | AGCAAAATGTTTGTGCCTGGG |
| *EGFR*-promotor BS4-F | ACCAAATTTGCAGCCCTTGT |
| *EGFR*-promotor BS4-R | GAATGCAGAGGCCATGAAGG |
| *EGFR*-promotor BS2-4-F | GTGAATGCACAGGACTTTATTGT |
| *EGFR*-promotor BS2-4-R | GGTTTTTCACCAGGGCAAGC |
| *EGFR*-promotor NS-F | TACAGCTGGCAAAGGGATGG |
| *EGFR*-promotor NS-R | CAGAGCCGTGTTTTACCCCT |

**Sup. Table S1. Primer Sequence**

**Sup. Table S2. Baseline Characteristics of the patients form GBM microarray**

| Sample No. | DNAJC10 expression Score | | | | OS Status | OS/Months | DFS Status | DFS/Months | Tumour grade |
| --- | --- | --- | --- | --- | --- | --- | --- | --- | --- |
|  | A | B | C | Median |  |  |  |  |  |
| P01A0626 | 2 | 3 | 3 | 3 | Living | 80 | DiseaseFree | 80 | I |
| P01A0659 | 3 | 4 | 4 | 4 | Living | 69 | DiseaseFree | 69 | I |
| P01A0601 | 4 | 6 | 4 | 4 | Living | 87 | DiseaseFree | 87 | I |
| P01A0567 | 6 | 6 | 6 | 6 | Living | 96 | DiseaseFree | 96 | I |
| P01A0614 | 6 | 6 | 6 | 6 | Living | 85 | DiseaseFree | 85 | I |
| P01A0646 | 6 | 8 | 9 | 8 | Living | 72 | DiseaseFree | 72 | I |
| P01A0654 | 6 | 8 | 6 | 6 | Living | 71 | DiseaseFree | 71 | I |
| P01A0656 | 6 | 6 | 6 | 6 | Living | 70 | DiseaseFree | 70 | I |
| P01A0573 | 8 | 9 | 8 | 8 | Living | 95 | DiseaseFree | 95 | I |
| P01A0600 | 8 | 8 | 8 | 8 | Living | 88 | DiseaseFree | 88 | I |
| P01A0609 | 8 | 8 | 8 | 8 | Living | 86 | DiseaseFree | 86 | I |
| P01A0623 | 8 | 8 | 8 | 8 | Living | 81 | DiseaseFree | 81 | I |
| P01A0687 | 8 | 9 | 9 | 9 | Living | 97 | DiseaseFree | 97 | I |
| P01A0512 | 9 | 12 | 9 | 9 | Living | 111 | DiseaseFree | 111 | I |
| P01A0538 | 9 | 9 | 9 | 9 | Living | 103 | DiseaseFree | 103 | I |
| P01A0542 | 9 | 9 | 9 | 9 | Living | 102 | DiseaseFree | 102 | I |
| P01A0545 | 9 | 6 | 8 | 8 | Living | 101 | DiseaseFree | 101 | I |
| P01A0627 | 9 | 8 | 6 | 8 | Living | 80 | DiseaseFree | 80 | I |
| P01A0507 | 12 | 12 | 12 | 12 | Living | 113 | DiseaseFree | 113 | I |
| P01A0517 | 12 | 8 | 8 | 8 | Living | 108 | DiseaseFree | 108 | I |
| P01A0536 | 12 | 12 | 12 | 12 | Living | 103 | DiseaseFree | 103 | I |
| P01A0539 | 12 | 12 | 8 | 12 | Living | 102 | DiseaseFree | 102 | I |
| P01A0547 | 12 | 12 | 12 | 12 | Living | 101 | DiseaseFree | 101 | I |
| P01A0595 | 2 | 2 | 2 | 2 | Deceased | 20 | Recurred | 20 | II |
| P01A0649 | 2 | 2 | 3 | 2 | Deceased | 26 | Recurred | 26 | II |
| P01A0653 | 3 | 3 | 3 | 3 | Living | 71 | DiseaseFree | 71 | II |
| P01A0564 | 4 | 4 | 4 | 4 | Deceased | 45 | Recurred | 45 | II |
| P01A0633 | 4 | 6 | 6 | 6 | Living | 77 | Recurred | 41 | II |
| P01A0635 | 4 | 6 | 4 | 4 | Living | 76 | Recurred | 76 | II |
| P01A0638 | 4 | 4 | 4 | 4 | Living | 76 | DiseaseFree | 76 | II |
| P01A0639 | 4 | 4 | 4 | 4 | Living | 75 | DiseaseFree | 75 | II |
| P01A0644 | 4 | 4 | 4 | 4 | Living | 74 | DiseaseFree | 74 | II |
| P01A0645 | 4 | 4 | 4 | 4 | Living | 74 | DiseaseFree | 74 | II |
| P01A0650 | 4 | 4 | 4 | 4 | Living | 71 | DiseaseFree | 71 | II |
| P01A0557 | 6 | 6 | 6 | 6 | Living | 99 | DiseaseFree | 99 | II |
| P01A0571 | 6 | 6 | 6 | 6 | Deceased | 57 | Recurred | 36 | II |
| P01A0581 | 6 | 6 | 6 | 6 | Deceased | 55 | Recurred | 47 | II |
| P01A0598 | 6 | 6 | 6 | 6 | Living | 88 | Recurred | 41 | II |
| P01A0603 | 6 | 6 | 6 | 6 | Living | 87 | Recurred | 53 | II |
| P01A0613 | 6 | 6 | 6 | 6 | Living | 85 | DiseaseFree | 85 | II |
| P01A0631 | 6 | 6 | 6 | 6 | Living | 77 | Recurred | 26 | II |
| P01A0636 | 6 | 6 | 6 | 6 | Living | 76 | DiseaseFree | 76 | II |
| P01A0544 | 8 | 8 | 9 | 8 | Living | 101 | DiseaseFree | 101 | II |
| P01A0550 | 8 | 8 | 8 | 8 | Living | 100 | Recurred | 63 | II |
| P01A0556 | 8 | 8 | 8 | 8 | Living | 99 | DiseaseFree | 99 | II |
| P01A0560 | 8 | 9 | 9 | 9 | Deceased | 59 | Recurred | 55 | II |
| P01A0578 | 8 | 8 | 8 | 8 | Living | 93 | Recurred | 13 | II |
| P01A0593 | 8 | 8 | 8 | 8 | Living | 89 | DiseaseFree | 89 | II |
| P01A0605 | 8 | 8 | 8 | 8 | Living | 87 | Recurred | 48 | II |
| P01A0607 | 8 | 6 | 6 | 6 | Deceased | 62 | Recurred | 44 | II |
| P01A0506 | 9 | 9 | 9 | 9 | Living | 113 | DiseaseFree | 113 | II |
| P01A0521 | 9 | 12 | 9 | 9 | Living | 108 | Recurred | 34 | II |
| P01A0527 | 9 | 9 | 9 | 9 | Living | 106 | Recurred | 86 | II |
| P01A0530 | 9 | 9 | 9 | 9 | Living | 105 | Recurred | 59 | II |
| P01A0531 | 9 | 9 | 9 | 9 | Living | 105 | DiseaseFree | 105 | II |
| P01A0535 | 9 | 9 | 8 | 9 | Living | 104 | DiseaseFree | 104 | II |
| P01A0548 | 9 | 9 | 12 | 9 | Living | 100 | DiseaseFree | 100 | II |
| P01A0552 | 9 | 9 | 9 | 9 | Living | 100 | DiseaseFree | 100 | II |
| P01A0563 | 9 | 9 | 9 | 9 | Living | 97 | Recurred | 55 | II |
| P01A0572 | 9 | 9 | 9 | 9 | Living | 96 | Recurred | 54 | II |
| P01A0575 | 9 | 9 | 9 | 9 | Living | 94 | DiseaseFree | 94 | II |
| P01A0616 | 9 | 9 | 9 | 9 | Living | 84 | DiseaseFree | 84 | II |
| P01A0516 | 12 | 12 | 12 | 12 | Living | 109 | DiseaseFree | 109 | II |
| P01A0518 | 12 | 12 | 12 | 12 | Living | 108 | DiseaseFree | 108 | II |
| P01A0524 | 12 | 8 | 12 | 12 | Deceased | 83 | Recurred | 83 | II |
| P01A0532 | 12 | 12 | 12 | 12 | Living | 105 | Recurred | 52 | II |
| P01A0533 | 12 | 9 | 12 | 12 | Living | 105 | DiseaseFree | 105 | II |
| P01A0569 | 12 | 12 | 12 | 12 | Living | 96 | Recurred | 50 | II |
| P01A0617 | 0 | 0 | 0 | 0 | Deceased | 11 | Recurred | 11 | III |
| P01A0574 | 1 | 1 | 1 | 1 | Deceased | 30 | Recurred | 30 | III |
| P01A0651 | 1 | 1 | 2 | 1 | Deceased | 11 | Recurred | 11 | III |
| P01A0629 | 2 | 1 | 2 | 2 | Deceased | 23 | Recurred | 23 | III |
| P01A0682 | 2 | 2 | 2 | 2 | Deceased | 19 | Recurred | 19 | III |
| P01A0608 | 3 | 3 | 3 | 3 | Deceased | 33 | Recurred | 33 | III |
| P01A0615 | 4 | 4 | 4 | 4 | Deceased | 32 | Recurred | 32 | III |
| P01A0630 | 4 | 4 | 6 | 4 | Deceased | 26 | Recurred | 26 | III |
| P01A0640 | 4 | 4 | 4 | 4 | Deceased | 18 | Recurred | 18 | III |
| P01A0655 | 4 | 4 | 4 | 4 | Deceased | 21 | Recurred | 21 | III |
| P01A0658 | 4 | 4 | 4 | 4 | Deceased | 37 | Recurred | 37 | III |
| P01A0546 | 6 | 6 | 6 | 6 | Deceased | 67 | Recurred | 81 | III |
| P01A0620 | 6 | 6 | 6 | 6 | Deceased | 27 | Recurred | 26 | III |
| P01A0549 | 8 | 8 | 8 | 8 | Living | 100 | Recurred | 46 | III |
| P01A0624 | 8 | 8 | 9 | 8 | Living | 81 | Recurred | 33 | III |
| P01A0555 | 9 | 9 | 9 | 9 | Deceased | 66 | Recurred | 58 | III |
| P01A0599 | 9 | 9 | 9 | 9 | Living | 88 | Recurred | 30 | III |
| P01A0621 | 9 | 8 | 9 | 9 | Living | 82 | DiseaseFree | 82 | III |
| P01A0510 | 12 | 12 | 12 | 12 | Living | 112 | Recurred | 27 | III |
| P01A0528 | 12 | 9 | 12 | 12 | Living | 105 | Recurred | 72 | III |
| P01A0667 | 0 | 2 | 2 | 2 | Deceased | 13 | Recurred | 13 | IV |
| P01A0670 | 0 | 0 | 0 | 0 | Deceased | 10 | Recurred | 10 | IV |
| P01A0678 | 0 | 0 | 0 | 0 | Deceased | 12 | Recurred | 12 | IV |
| P01A0680 | 0 | 0 | 1 | 0 | Deceased | 6 | Recurred | 6 | IV |
| P01A0683 | 1 | 1 | 0 | 1 | Deceased | 9 | Recurred | 9 | IV |
| P01A0684 | 1 | 0 | 1 | 1 | Deceased | 10 | Recurred | 10 | IV |
| P01A0662 | 1 | 1 | 0 | 1 | Deceased | 12 | Recurred | 12 | IV |
| P01A0685 | 2 | 2 | 2 | 2 | Deceased | 14 | Recurred | 14 | IV |
| P01A0677 | 3 | 3 | 2 | 3 | Deceased | 17 | Recurred | 17 | IV |
| P01A0681 | 3 | 3 | 3 | 3 | Deceased | 10 | Recurred | 10 | IV |
| P01A0668 | 4 | 3 | 4 | 4 | Deceased | 11 | Recurred | 11 | IV |
| P01A0663 | 6 | 4 | 6 | 6 | Deceased | 22 | Recurred | 22 | IV |
| P01A0666 | 6 | 6 | 6 | 6 | Deceased | 50 | Recurred | 41 | IV |
| P01A0673 | 6 | 4 | 6 | 6 | Deceased | 32 | Recurred | 32 | IV |
| P01A0676 | 6 | 4 | 4 | 4 | Deceased | 34 | Recurred | 23 | IV |
| P01A0665 | 8 | 6 | 6 | 6 | Deceased | 33 | Recurred | 33 | IV |
| P01A0669 | 8 | 8 | 8 | 8 | Deceased | 15 | Recurred | 12 | IV |
| P01A0671 | 8 | 6 | 6 | 6 | Deceased | 23 | Recurred | 23 | IV |
| P01A0674 | 9 | 8 | 9 | 9 | Deceased | 41 | Recurred | 34 | IV |

**Sup. Table S3. UPR related genes and Candidate EGFR transcription factors**

| UPR related genes from PathCards | Candidate EGFR transcription factors from PROMO |
| --- | --- |
| ACADVL, ADD1, ARFGAP1, ASNS, ATF3, ATF4, ATF6, ATP6V0D1, CALR, CCL2, C/EBPβ, C/EBPγ, CREB3, CREB3L1, CREB3L2, CREB3L3, CREB3L4, CREBRF, CTDSP2, CUL7, CXCL8, CXXC1, DCP2, DCSTAMP, DCTN1, DDIT3, DDX11, DIS3, DNAJB11, DNAJB9, DNAJC3, EDEM1, EIF2AK3, EIF2S1, EIF2S2, EIF2S3, ERN1, EXOSC1, EXOSC2, EXOSC3, EXOSC4, EXOSC5, EXOSC6, EXOSC7, EXOSC8, EXOSC9, EXTL1, EXTL2, EXTL3, FKBP14, GFPT1, GOSR2, GSK3A, HDGF, HERPUD1, HSP90B1, HSPA5, HYOU1, IGFBP1, JMJD7-PLA2G4B, KDELR3, KHSRP, KLHDC3, LMNA, MBTPS1, MBTPS2, MYDGF, NF-Y, NFYB, NFYC, PARN, PDIA5, PDIA6, PLA2G4B, PPP2R5B, PREB, SEC31A, SERP1, SHC1, SRPRA, SRPRB, SSR1, SULT1A3, SULT1A4, SYVN1, TATDN2, TLN1, TPP1, TSPYL2, WFS1, WIPI1, XBP-1, YIF1A, ZBTB17 | TFAP2A, TFAP2B, AR, ATF3, CEBPα, C/EBPβ, C/EBPγ, RXRA, ETS1, ETS2, JUN, MYB, NR2F1, CRX, E2F1, EBF1, EGR1, EGR3, ELK1, ESR1, FOXF1, FOXP3, GATA1, GATA2, GCFC2, NR3C1, HES1, HNF1A, HNF1B, FOXA1, FOXA2, HNF4A, HOXD10, HOXD9, IKZF1, IRF1, IRF2, LEF1, MAZ, MYOD1, NRF1, NFATC2, NFATC1, NFI/CTF, NFKB1, NF-Y, TP53, PAX5, ETV4, POU1F1, POU2F1, POU2F2, PGR, SPI1, RARB, RBPJ, RELA, SP1, SREBF1, SRY, STAT1, STAT4, STAT5A, TCF4, TBP, GTF2I, USF1, USF2, VDR, WT1, XBP-1, YY1 |

**Sup. Table S4. Candidate XBP-1s binding sites at the EGFR promoter region**

| Factor name | Positions | Dissimilarity | String | RE equally | RE query |
| --- | --- | --- | --- | --- | --- |
| XBP-1s [T00902] | (-1630 to -1625) | 11.373637 | ATGGCC | 1.953 | 1.935 |
| XBP-1s [T00902] | (-1597 to -1592) | 6.478682 | GGGCAT | 0.976 | 1.004 |
| XBP-1s [T00902] | (-1593 to -1588) | 0.000000 | ATGACT | 0.976 | 0.931 |
| XBP-1s [T00902] | (-1099 to -1094) | 6.478682 | TGGCAT | 0.976 | 1.004 |
